# Reduction of Redox Potential Exerts a Key Role in Modulating Gut Microbial Taxa and Function by Dietary Supplementation of Pectin in a Pig Model

**DOI:** 10.1128/spectrum.03283-22

**Published:** 2022-12-08

**Authors:** Rongying Xu, Qiuke Li, Hongyu Wang, Yong Su, Weiyun Zhu

**Affiliations:** a Laboratory of Gastrointestinal Microbiology, Jiangsu Key Laboratory of Gastrointestinal Nutrition and Animal Health, College of Animal Science and Technology, Nanjing Agricultural University, Nanjing, China; b National Center for International Research on Animal Gut Nutrition, Nanjing Agricultural University, Nanjing, China; Jilin University

**Keywords:** pectin, pig model, gut microbiota, inosine, multi-omics, redox potential

## Abstract

Pectin exists in a vast range of plants and has a long history of acting as a functional food additive with potential prebiotic effects on intestinal health. However, knowledge of how pectin regulates gut microbial communities is still insufficient and limited. Here, metatranscriptome sequencing revealed that a pectin-enriched diet (PEC) decreased the abundances of fungal keystone taxa (e.g., amino acid-producing *Kazachstania* spp.) and their genes involved in oxidative phosphorylation, while it increased the abundance of sulfate-reducing *Desulfovibrio* spp., and methane-producing *Methanobrevibacter* spp. in colon microbiomes. Furthermore, we first confirmed that PEC decreased fecal redox potential in a fistula pig model, which could be supported by the enrichment of antioxidants (e.g., inosine) in feces. Fecal metagenome analysis disclosed that certain microbial taxa promoted inosine biosynthesis from pectin degradation, including *Prevotella*, which plays an essential role in pectin biodegradation. Overall, these results demonstrate that pectin decreases the redox potential in pig hindgut to modulate microbial composition and functions, and specific microorganisms generate reducing agents in the course of pectin degradation to decrease redox potential of microbial ecosystem.

**IMPORTANCE** Collective studies indicate that pectin degradation promotes extensive microorganisms that can be involved in pectin degradation directly or indirectly, or benefit from the altered physiological conditions caused by pectin ingestions. Our study focuses on effects of pectin on gut microbial taxa and functions, as well as its interactions with altered environmental features. Our results demonstrate pectin-induced proreducing shifts on colon microbial taxa and functions, and first confirm that pectin decreases hindgut redox potential, which is an important environmental feature that can modulate microbial communities. These results infer that there is bidirectional regulation between microbiota and redox potential during pectin degradation. In general, this investigation proposes new insights into the pectin-modulating gut microbial ecosystem and also provides new perspectives for targeting modulation of gut microbiota.

## INTRODUCTION

In mammals, the gastrointestinal tract harbors trillions of microbes that are essential for host physiology and metabolism (1). Manipulation of gut microbiota appears to be a promising approach to improve host health. Dietary fiber such as pectin has been explored as a tool to modulate gut microbiota composition (2, 3). Pectin is a complex polysaccharide that contains galacturonic acid (GalA) units with α-1,4 linkages as the main chain and sugars (e.g., rhamnose, arabinose, and others) as the branches. Major pectin remains intact during its transit through the upper gastrointestinal tract and reaches the hindgut to be fermented by microorganisms (4). The anaerobic condition in hindgut promotes the production of fermentation products. Prior studies have assessed the impact of pectin ingestion on the microbial community (5 to 7). However, there is little systemic knowledge about how pectin regulates gut microbiota.

Gut luminal pH and redox state are important environmental features indicating the stability of the microbial ecosystem (8 to 10). However, unlike pH, which has been generally discussed, the effect of the redox state on gut microbial communities is generally ignored. In fact, the changes of redox status can modulate metabolic types in the microbial community and allow specific microbes to thrive (11, 12). For example, as reported by Friedman et al. (13), redox potential changed with diet and drove compositional shifts in the rumen archaeal community in the abundance of methanogenic archaea. Therefore, it is promising to develop regulation strategies targeting gut microbiota based on redox potential.

It is vital for gut homeostasis to maintain a low gut redox potential in an anaerobic environment through dietary intervention (14). Under antibiotics exposure or in the case of disease (e.g., obesity, malnutrition, and intestinal damage), the normal gut microbial ecosystem will always be disrupted, coupled with increased redox potential (10, 15). Hopefully, these can be alleviated through antioxidant supplementation in food (16). Previous studies have reported the ability of dietary fibers to improve host health by modulation of the redox balance, and observed that antioxidant dietary fiber induced a proreducing shift in the redox state of the colonic mucosa (17). Observational studies confirmed the inverse correlation between fruit and vegetable (rich in dietary fiber) intake and oxidative stress markers, suggesting its potential to improve gut redox state (17, 18). Nevertheless, whether pectin has similar potential to improve gut redox status remains unknown.

So far, the regulatory role of pectin on gut microbial taxa and function, and corresponding underlying mechanisms, remains unclear. Here, we performed metatranscriptomics analysis to explore the modulation of pectin on colon microbial taxa and function, and developed pectin intervention experiments *in vitro* and *in vivo*, together with multi-omics analysis in a fistula pig model to explore potential regulatory mechanisms of pectin on gut microbiome. These findings propose new insights into pectin modulating gut microbial ecosystems, and also provide new perspectives for targeting modulation of gut microbiota.

## RESULTS

### Pectin-enriched diet (PEC) modulated colon microbial structure at the RNA level.

First, eight samples were randomly selected for colon metatranscriptome sequencing, and generated 98 Gb of paired-end sequencing data, which contained an average of 12.3 Gb (11.2 to 13.7 Gb) per sample. In total, 595,267 contigs were assembled with an average N_50_ length of 1.95 kb, including an average length of open reading frame (ORF) of 827 bp.

**FIG 1:**
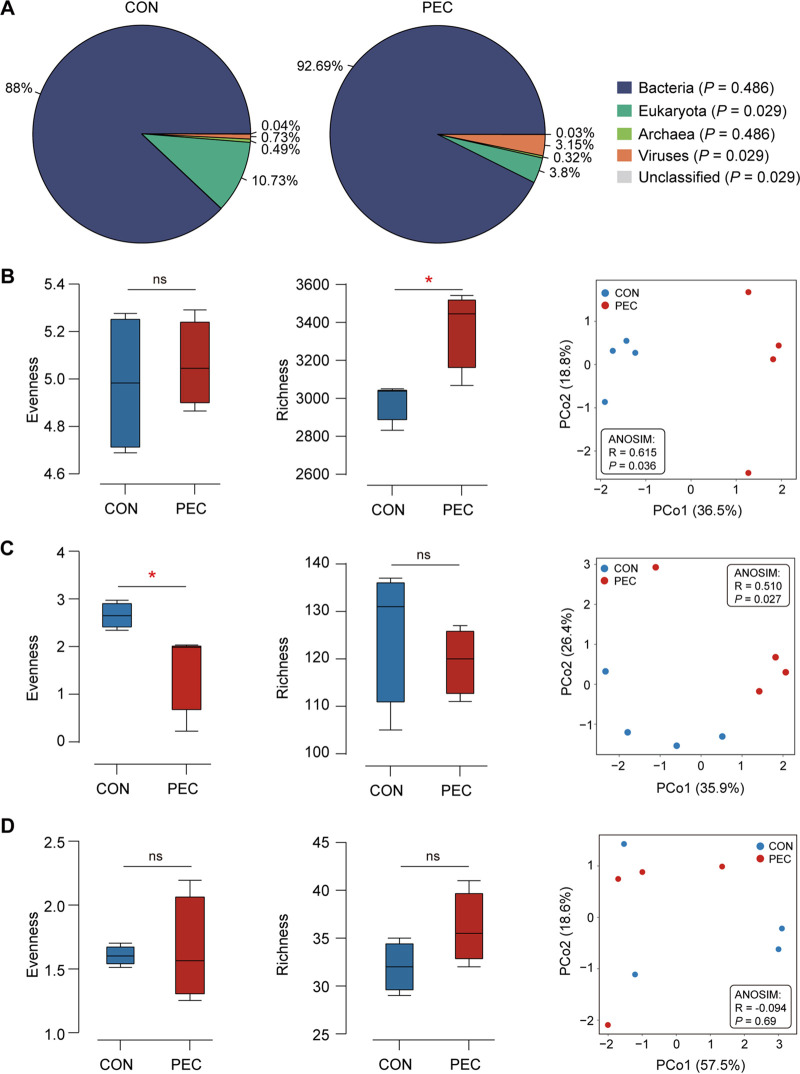
PEC changed colon microbial structure at the RNA level in pigs. (A) Microbial composition at the domain level. Effects of PEC on bacterial (B), fungal (C), and archaeal (D) diversity in pig colons. The evenness (Shannon index) and richness (number of observed species) were compared between the two groups (*n *= 4 per group). *, *P < *0.05; ns, *P > *0.05. Principal coordinate analysis (PCoA) was performed based on the Bray-Curtis metric and an ANOSIM test.

Compared with the control diet (CON) group, the proportions of Eukaryota and viruses were significantly decreased in the PEC group (*P < *0.05) (Fig. 1A). Besides, the bacterial richness was significantly increased by PEC (Fig. 1B), while the species evenness (Shannon index) of fungi was significantly decreased (*P < *0.05; Fig. 1C). The PCoA plots also showed clear segregations and dissimilarities between the CON and PEC groups based on bacterial (Fig. 1B) and fungal (Fig. 1C) species (*P < *0.05). However, no significant difference was observed in archaea (*P > *0.05; Fig. 1D).

**FIG 2:**
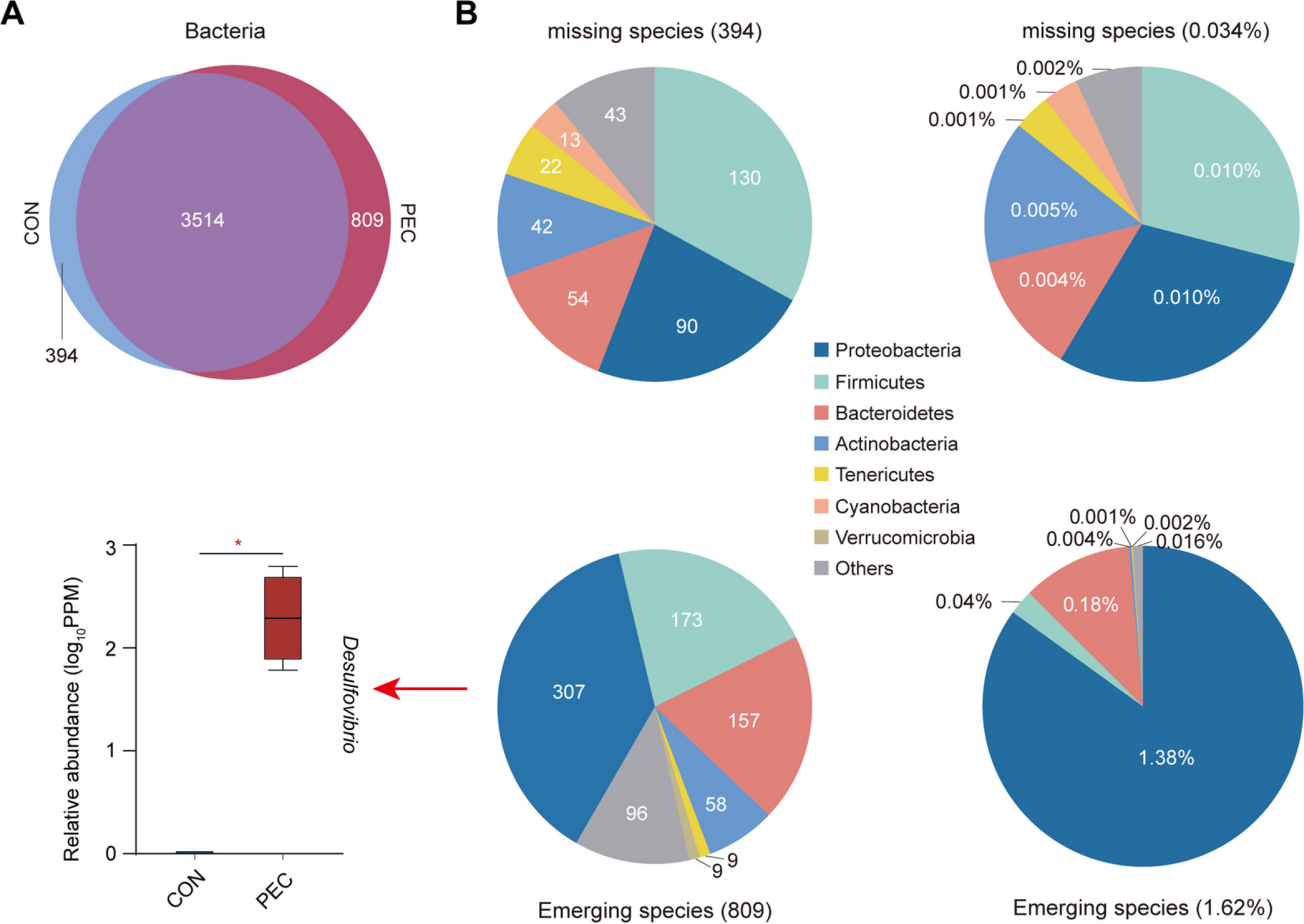
(A) Venn plot based on the species level of bacteria in the colon of pigs (*n *= 4 per group). (B) The number and proportion of missing and emerging species of bacteria in the PEC group. The parts with different colors represent the different taxonomic distribution of species at the phylum level. The significance test based on the relative abundance of *Desulfovibrio* is shown on the left. *, *P < *0.05.

Then, we identified all bacterial species and found that 3,514 species were shared between the two groups (Fig. 2A). As shown in Fig. 2B, 307 emerging species assigned to Proteobacteria occupied 1.38% of all species, but missing species only accounted for 0.01%. Particularly, the relative abundance of *Desulfovibrio* was higher (*P < *0.05) in the PEC group.

### PEC changed composition and keystone taxa of colon fungi.

**FIG 3:**
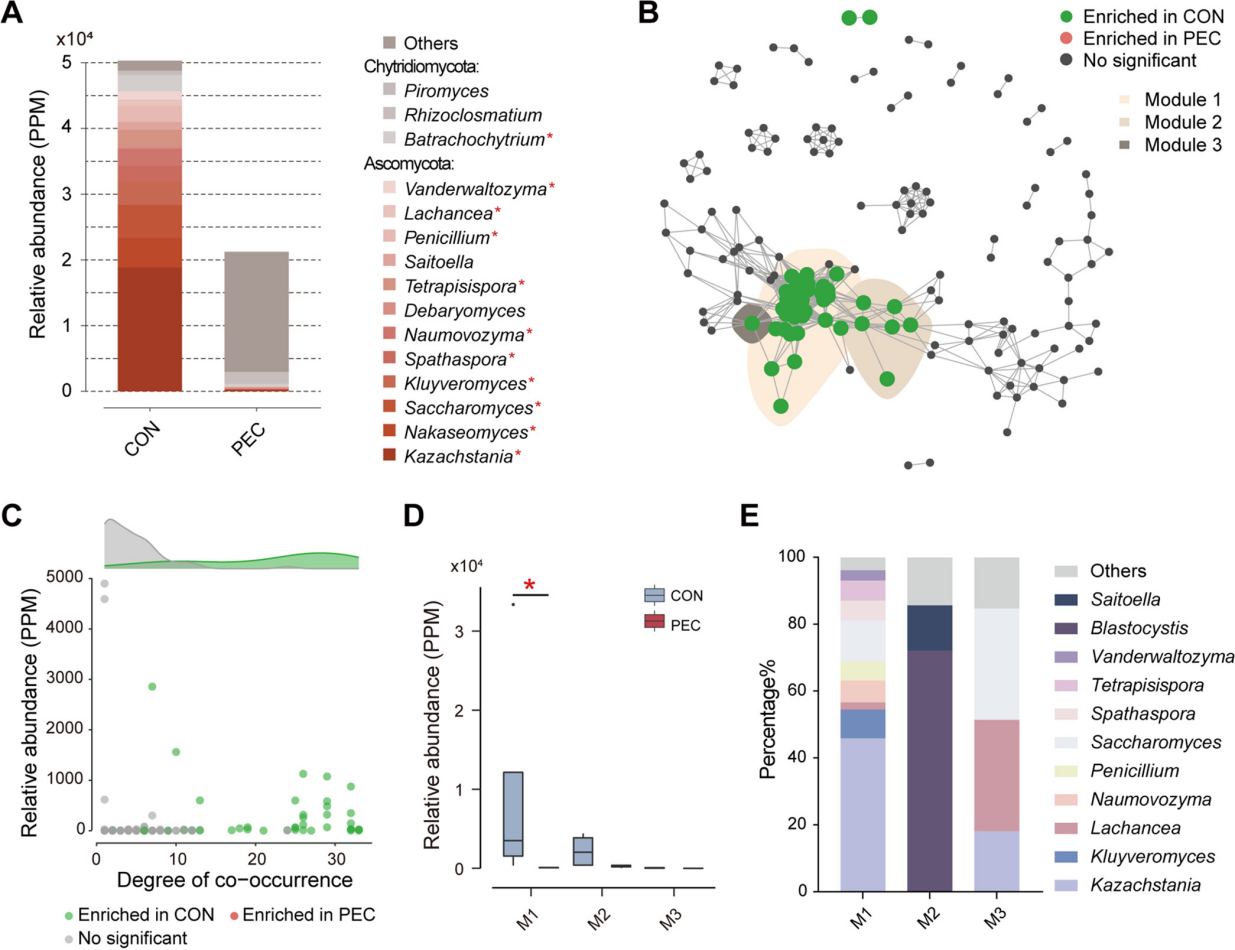
PEC-altered composition and keystone taxa of colon fungi in pigs. (A) Dominant fungal genera of more than 0.5% at least in one group (*n *= 4 per group). Mann-Whitney *U* test, *, *P < *0.05. (B) Cooccurrence network of microbial species. Cooccurrence networks visualizing significant correlations (*rho* > 0.7, *P < *0.001; indicated with gray lines) between fungal species. Circles represent fungal species. Species circles are colored in green (enriched in CON) or red (enriched in PEC). Shaded areas represent the network modules containing species with significant difference between the two groups. (C) Degree of cooccurrence of all species in the cooccurrence network. Colored circles represent that the species are enriched in CON (green) and PEC (red) groups, or have no significant difference between the two groups. (D) Cumulative relative abundances (parts per million, PPM) of fungal modules. Mann-Whitney *U* test, *, *P < *0.05. (E) Taxonomic composition of the treatment-sensitive modules at the genus level.

At the genus level, we found that the abundances of 11 dominant fungal genera (consisting of *Kazachstania*, *Nakaseomyces*, *Batrachochytrium*, etc.) were all significantly lower in the colon of PEC pigs (*P < *0.05; Fig. 3A; Table S1 in the supplemental material). At the species level, the abundances of the top 15 fungal species (including *Kazachstania naganishii*) were all lower in the colon of PEC pigs (*P < *0.05; Fig. S1). Then, we constructed a separate cooccurrence network for colon fungal species (Fig. 3B). In this network, three modules that contained dissimilatory cooccurring species with higher relative abundances than the other group were noted. Correspondingly, network connectivity (measured by average number of connections per species) and complexity of modules where differential cooccurring fungal species existed were higher than that of other modules in this network, suggesting the keystone taxa of fungi were affected by PEC. The cooccurrence degrees of each node (number of connections to a node) were measured and showed that species enriched in the CON group had medium to high degrees of cooccurrence (Fig. 3C). The cumulative relative abundance of Module 1 (M1) was significantly lower in the PEC group compared with the CON group (*P < *0.05; Fig. 3D). The phylogenetic distribution of the three modules displayed that species in M1 were assigned to a wider range of fungi than the other two modules (Fig. 3E).

### Functional profiles of the colon microbiome and differential functions between the CON and PEC pigs.

To evaluated the functional activities of the colon microbiome, we identified a total of 3,649 KEGG Orthology (KO) genes (74 upregulated and 475 downregulated by PEC), and 220 endogenous third-level pathways were identified in these samples (Table S2). Among them, 432 genes displayed a ≥5-fold difference in expression between the two groups of samples. Pathway enrichment analysis revealed that most differentially expressed genes (DEGs) were catalogued into 11 metabolic pathways (Table 1), and strikingly, almost all DEGs involved in oxidative phosphorylation exhibited lower abundances upon PEC.

**TABLE 1:** Pathway enrichment of DEGs (differentially expressed genes) with great (>5-fold) differences in relative abundance between the CON and PEC groups (*n *= 4 per group)^*a*^

Pathway	*P* value	Count of DEGs				Total
		Upregulated		Downregulated		
		5 to 20	>20	5 to 20	>20	
Oxidative phosphorylation	0.029*	0	0	3	25	28
Carbon metabolism	0.343	1	21	1	4	27
Methane metabolism	0.029*	1	23	0	1	25
Biosynthesis of amino acids	0.686	1	1	1	12	15
Ribosome	0.029*	0	0	3	11	14
Nucleocytoplasmic transport	0.029*	0	0	0	11	11
ABC transporters	1.000	0	4	3	1	8
Pyrimidine metabolism	0.114	0	1	3	1	5
Nicotinate and nicotinamide metabolism	0.114	0	3	0	2	5
Purine metabolism	0.343	1	0	2	2	5
Butanoate metabolism	0.029*	0	0	1	2	3

a The hypergeometric test was used to examine enrichment of pathways against the Kyoto Encyclopedia of Genes and Genomes (KEGG) database. *, *P *< 0.05. The counts of DEGs with fold change ranging from 5 to 20 and >20 in each pathway are listed.

### PEC repressed energy metabolism and gene expression processes in colon fungi.

**FIG 4:**
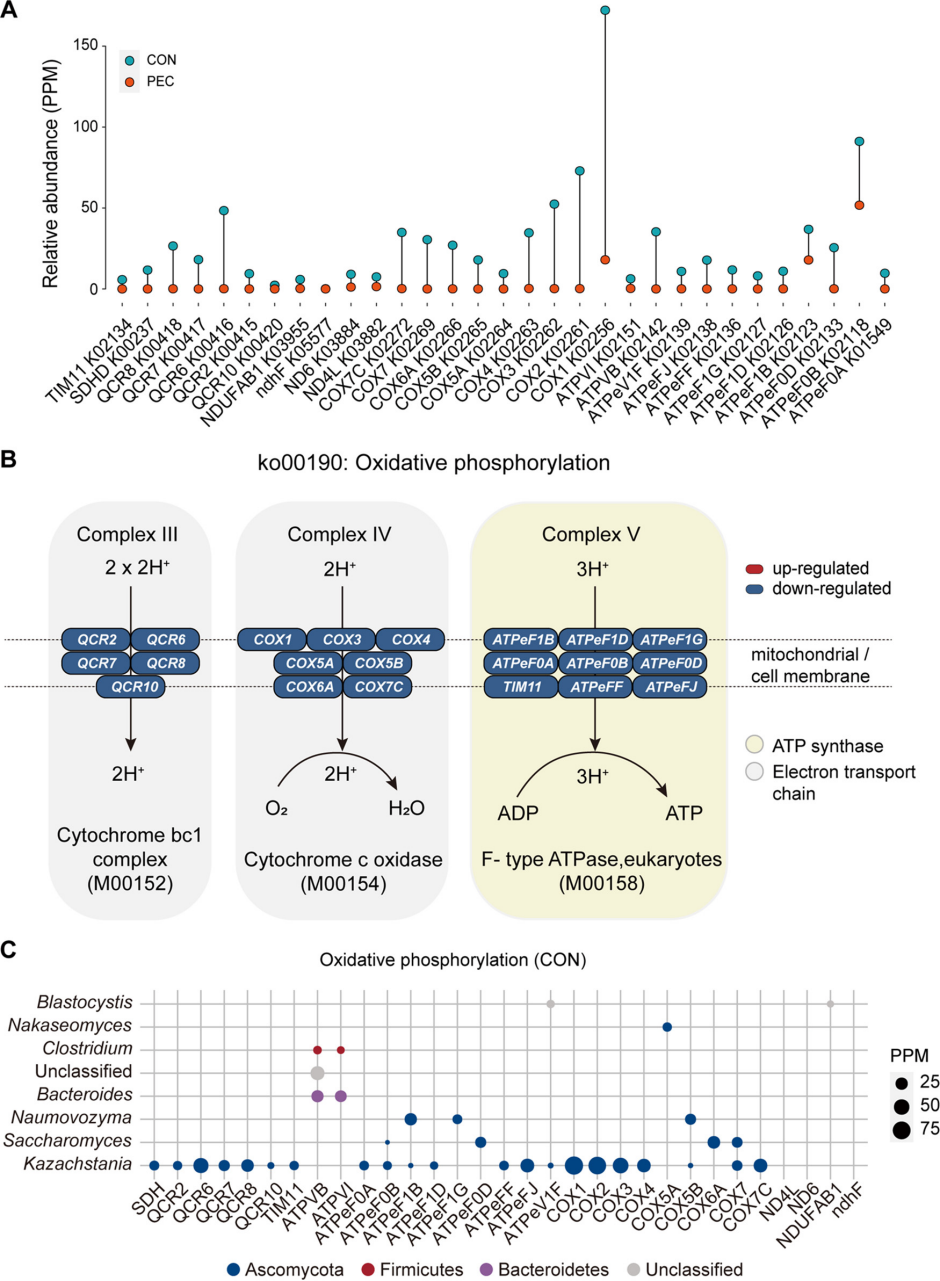
Microbial functions and genera involved in oxidative phosphorylation in the CON and PEC groups. (A) Comparison of the relative abundance of KO genes related to the oxidative phosphorylation pathway of pigs in the two groups (*n *= 4 per group). (B) Metabolic routes for oxidative phosphorylation (ko00190) containing electron transport chain (shaded in gray area) and ATP synthase (shaded in yellow area). The ellipses’ color represents two kinds of regulation by pectin-enriched diet. Blue, downregulation; red, upregulation. (C) Phylogenetic distribution of KO genes related to oxidative phosphorylation (ko00190) in the CON group at the genus level. The circle size means the relative abundance (PPM) of genes.

The relative abundances of genes involved in oxidative phosphorylation (Fig. 4A) were demonstrated and further exhibited in a pathway representation (Fig. 4B). Our findings showed that all shifted genes encoding proteins that play a role in the electron transport chain (including cytochrome bc_1_ complex/M00152 and cytochrome c oxidase/M00154 modules) and ATP synthase (F-type ATPase, eukaryotes/M00158) showed lower abundance upon PEC.

Phylogenetic distribution analysis revealed that genes with higher relative abundance in the CON group were mainly assigned to fungal genera (Fig. 4C). In parallel, *Kazachstania*, *Lingulodinium*, *Saccharomyces*, and *Naumovozyma* were the most assigned genera for the majority of genes enriched in the CON group. Moreover, many genes involved in the mRNA surveillance pathway, nucleocytoplasmic transport, and ribosome, mainly assigned to fungal genera, were downregulated in the colon of PEC pigs (Fig. S2).

### PEC altered carbon metabolism and amino acid biosynthesis in the colon microbiome of pigs.

**FIG 5:**
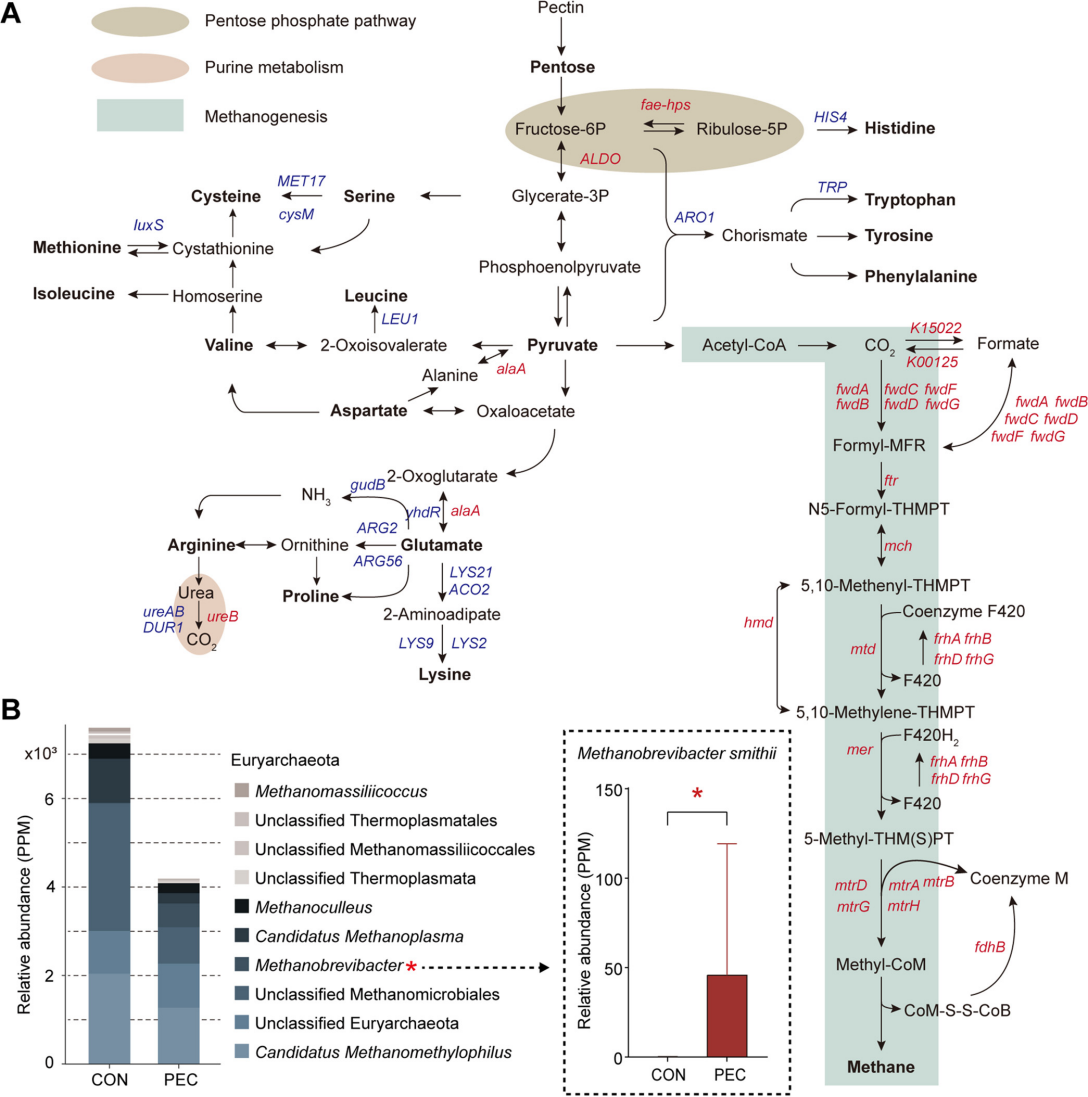
PEC changed carbon metabolism and amino acid metabolism in colon metatranscriptome. (A) Comparisons of the relative abundance of KO enzymes involved in the pentose phosphate pathway, methanogenesis, and amino acid biosynthesis between the CON and PEC groups (Mann-Whitney *U* test). Red font indicates upregulated enzyme genes, while blue font indicates downregulated enzyme genes. (B) Dominant archaeal genera of more than 0.5% at least in one group. Mann-Whitney *U* test, *, *P < *0.05.

To explore the effects of PEC on microbial functional differences involved in nutrient metabolism, we analyzed colon microbial carbon metabolism and amino acid biosynthesis pathways according to metatranscriptome information (Fig. 5A). Results showed that KO enzyme genes involved in amino acid biosynthesis were extensively downregulated upon PEC, while genes, including *fae-hps* (EC: 4.1.2.43) and *ALDO* (EC: 1.1.3.41) involved in the pentose phosphate pathway, through which pectin was converted into a mixture of 3- to 7-carbon sugar phosphates, were upregulated upon PEC. Thus, many genes involved in methanogenesis (converted from CO_2_ to methane) were upregulated in PEC pigs (Fig. S3). Taxonomic composition analysis of archaea at the genus level showed that the abundance of genus *Methanobrevibacter* (including Methanobrevibacter smithii) was obviously increased upon PEC (*P < *0.05; Fig. 5B). Methanobrevibacter smithii is an H_2_-utilizing archaea, which is vulnerable to trace amounts of free radicals, surviving only in a strongly anaerobic environment.

### PEC decreased redox potential associated with its metabolic process *in vivo*.

**FIG 6:**
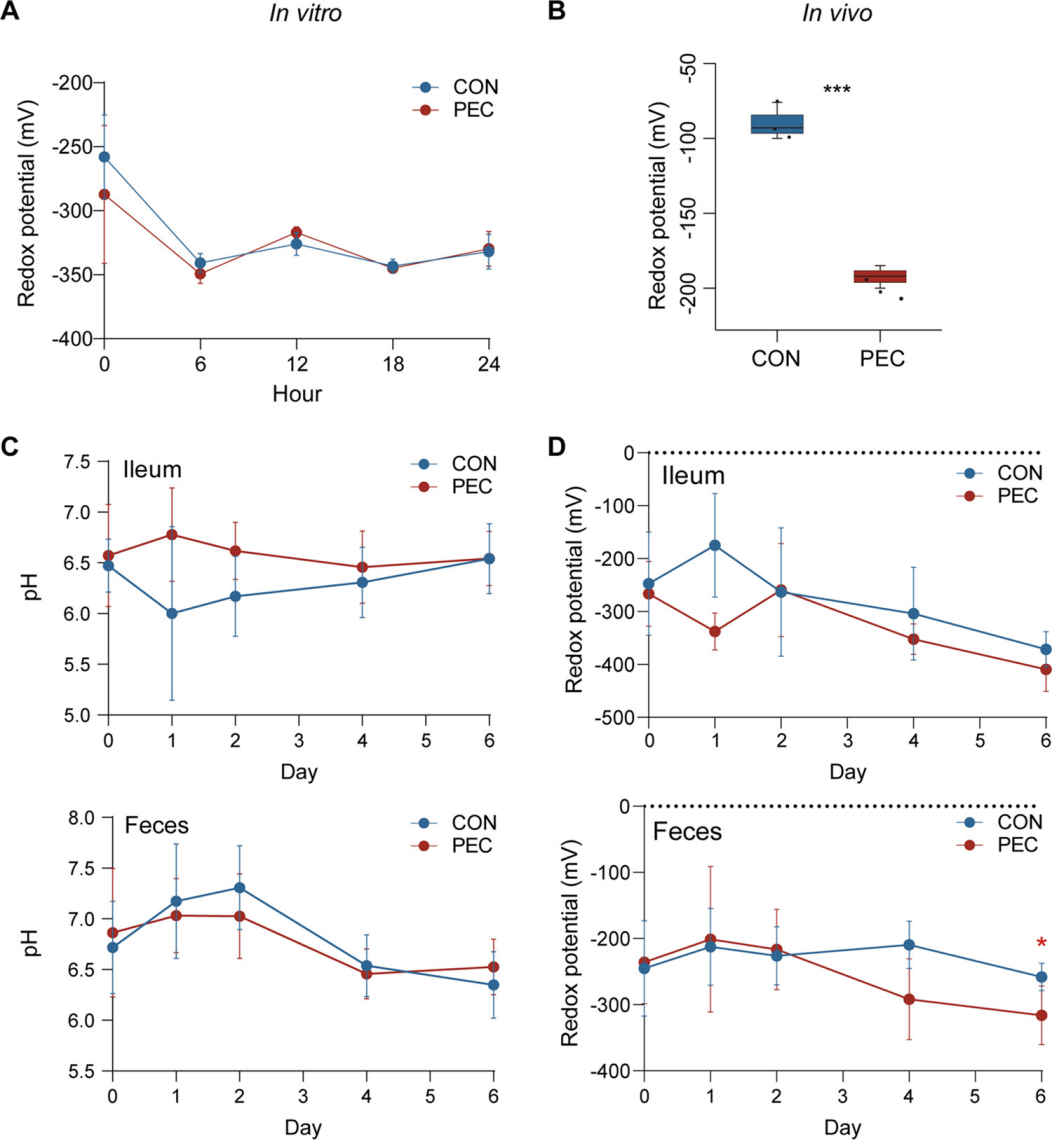
PEC decreased redox potential associated with its metabolic process *in vivo*. (A) Comparison of redox potential of *in vitro* cultivation between the two groups. (B) Comparison of redox potential in feces of growing pigs after 6 days with PEC treatment (Student's *t* test, ***, *P < *0.001). (C) Comparison of pH value in ileum contents and feces between the two groups. (D) Comparison of redox potential in ileum contents and feces between the two groups (Student's *t* test, *, *P < *0.05).

Regarding microorganisms that have different preferences in redox conditions (13, 19), we speculated that PEC altered hindgut luminal redox conditions mirrored by changes in the colon microbiome, as described above. To test this hypothesis, we measured the redox potential in *in vitro* and *in vivo* experiments. First, an *in vitro* cultivation experiment was performed to simulate the biodegradation process of pectin in pig colons, and the indicated pectin failed to change redox potential during cultivation (*P > *0.05; Fig. 6A). However, in an *in vivo* experiment, feeding with pectin for 6 days significantly decreased the fecal redox potential in growing pigs (*P < *0.01; Fig. 6B). Then, a fistula pig model was applied to investigate the environmental features dynamics, including pH (Fig. 6C) and redox potential (Fig. 6D), in the ileum and feces under the PEC treatment. Notably, at day 6, fecal redox potential was significantly decreased by PEC (*P < *0.05).

### PEC changed the metabolic profile both in ileum contents and feces of fistula pigs.

**FIG 7:**
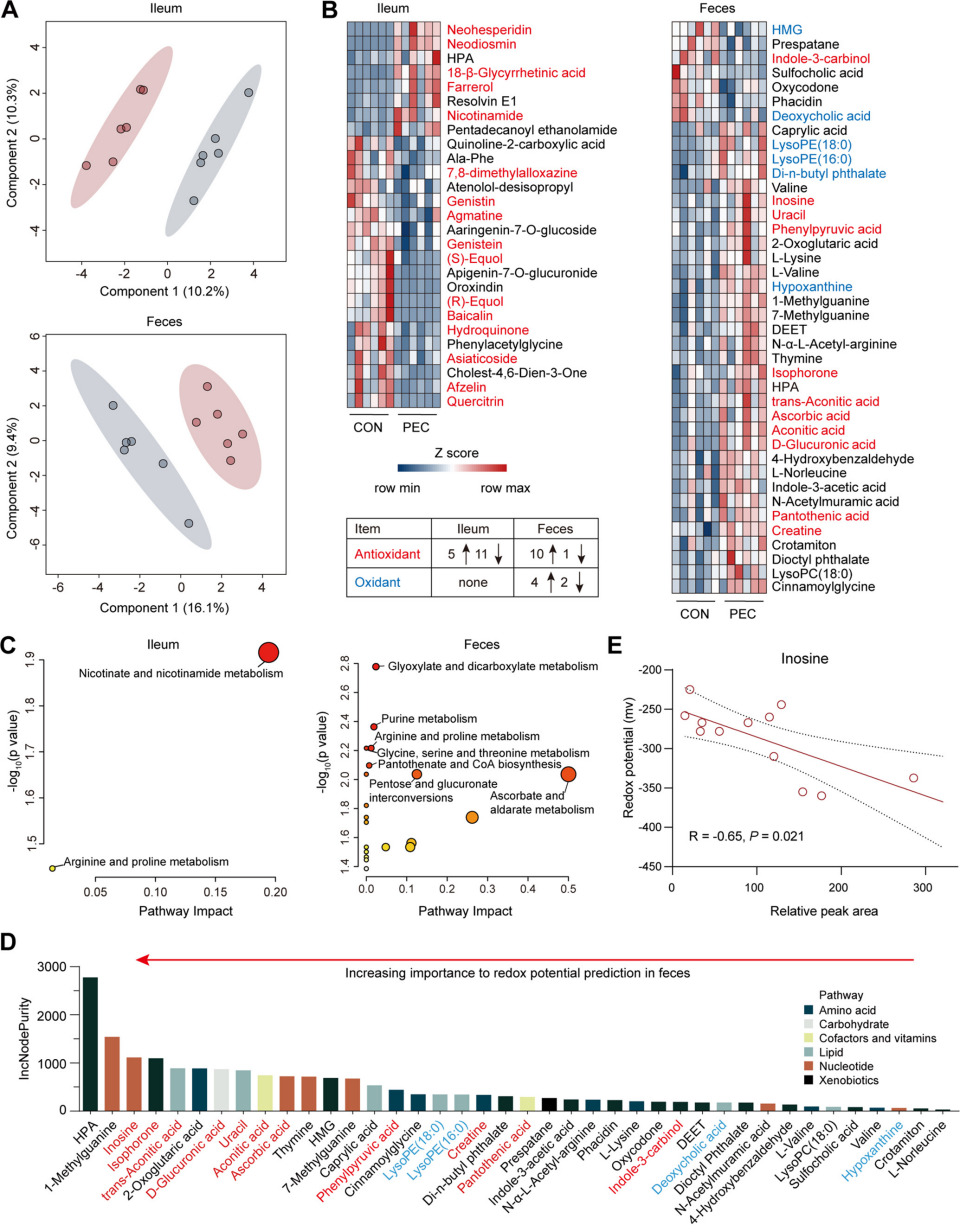
Pectin-enriched diet altered metabolomic characteristics in both ileal contents and feces of fistula pigs. (A) Sparse partial least-squares discriminant analysis (sPLS-DA) of metabolomic profiles in ileal contents and feces of CON and PEC groups (*n *= 6 per group). (B) Heat map of significantly altered metabolites (Student's *t* test, *P < *0.05) in ileal contents and feces of CON and PEC groups (*n *= 6 per group). Red font indicates antioxidant; blue font indicates oxidant. The direction of arrows indicates the up- or downregulation. (C) Pathway enrichment analysis performed using the significantly different fecal metabolites between the CON and PEC groups. (D) Significantly different metabolites were ranked in descending order of importance to redox potential prediction using random forest regression. Red font indicates antioxidant; blue font indicates oxidant. (E) The correlation between inosine and redox potential in pig feces. The Spearman’s rho with asymptotic measure-specific *P* value between inosine and redox potential was calculated using the hmisc R package (v.4.2.0), and *P < *0.05 was used to identify a significant correlation.

Untargeted metabolomic profiles were generated on ileal contents and feces to assess metabolic variations in response to PEC at day 6. The sparse PLS discriminant analysis (sPLS-DA) plot exhibited a significant separation of clusters between the CON and PEC groups (Fig. 7A). PEC significantly altered 27 metabolites (8 upregulated and 19 downregulated) and 40 metabolites (33 upregulated and 7 downregulated) in pig ileal contents and feces, respectively (Fig. 7B, Table S3). Taking the results of redox potential into consideration, we speculated that the abundances of antioxidants and oxidants, which can influence the redox status in the gut (20), might be modulated by PEC. After a bibliography search, we identified that 16 antioxidants (5 upregulated and 11 downregulated) and no oxidant in the ileal contents, and 11 antioxidants (10 upregulated and 1 downregulated) and 6 oxidants (4 upregulated and 2 downregulated) in the feces (Fig. 7B), were altered by PEC. Based on altered metabolites, metabolic pathway analysis (MetPA) exhibited the enrichments of 2 pathways (nicotinate and nicotinamide metabolism, and arginine and proline metabolism) and 24 pathways (including glyoxylate and dicarboxylate metabolism and purine metabolism) in ileal contents and feces, respectively (Fig. 7C).

Random forest regression analysis revealed that antioxidants dominated the top important predictors (metabolites) of fecal redox potential (Fig. 7D), and inosine, which is an essential intermediate metabolite involved in purine metabolism, was the most important antioxidant in predicting the fecal redox potential in response to PEC. Specifically, the Spearman rank correlation analysis between the scaled abundance of inosine and fecal redox potential revealed that inosine and fecal redox potential had a negative correlation (*R* = −0.65, *P = *0.021) (Fig. 7E).

### PEC promoted microbiota-derived inosine biosynthesis in feces.

**FIG 8:**
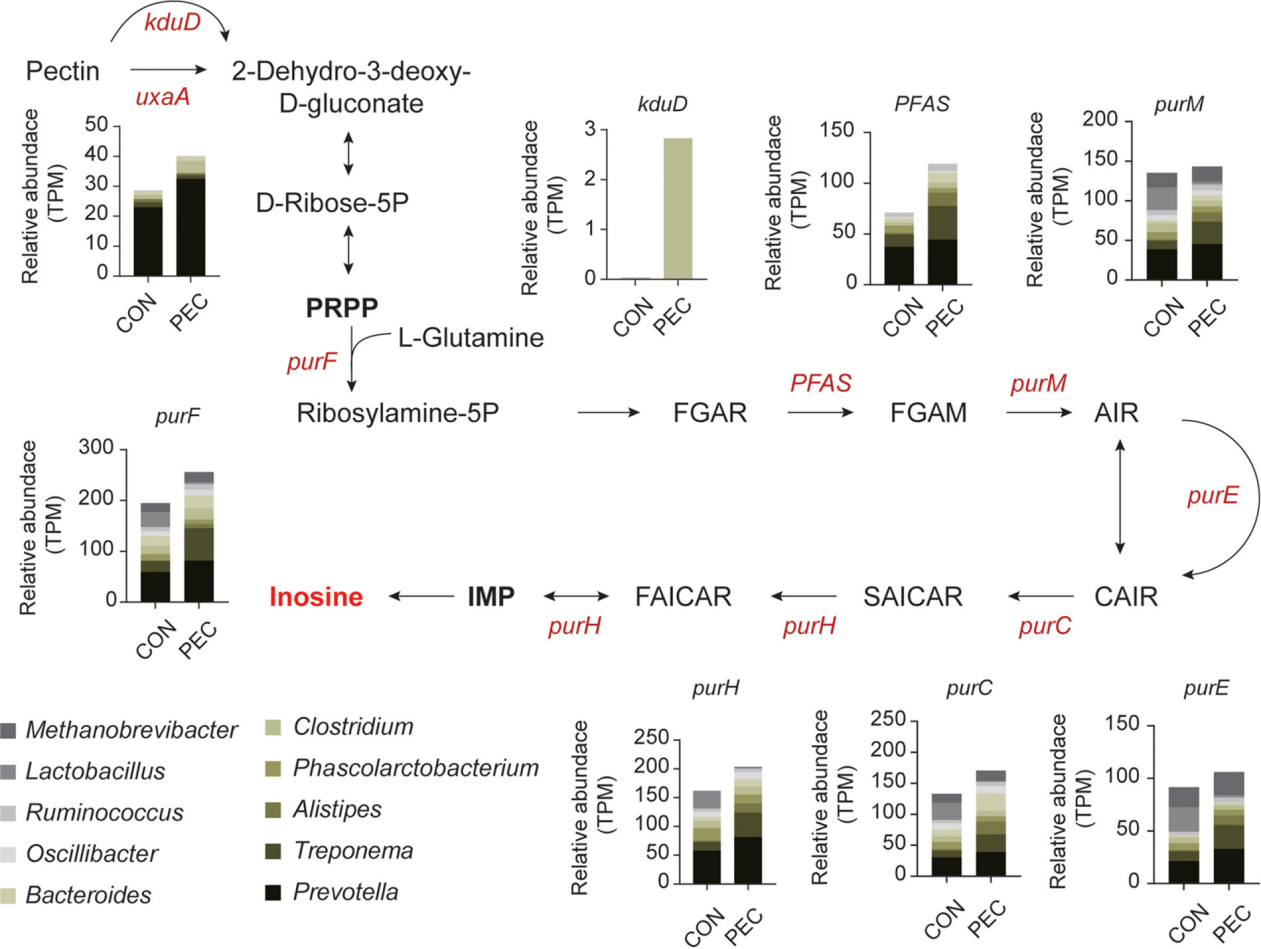
PEC changed inosine biosynthesis in fecal microbiota according to metagenome information. The relative abundance of KO enzymes involved in inosine biosynthesis was compared using the Mann-Whitney *U* test (*n *= 4 per group). Red font indicates upregulated enzyme genes. Stacked bars represent the phylogenetic distribution of differential KO enzyme genes.

Metagenome sequencing generated a total of 813,900,306 reads, with 101,737,538 ± 7,540,256 (mean ± standard error of the mean [SEM]) reads per sample. After quality filtering and host-genome removal, a total of 729,490,352 reads were retained, with 91,186,294 ± 7,011,375 per sample. To explore why inosine upregulated by PEC, we constructed the metabolic route for inosine biosynthesis directly converted from pectin according to metagenome information. In this study, we found that following the transformation from pectin to phosphoribosyl diphosphate (PRPP), *uxaA* (EC: 4.2.1.7) and *kduD* (EC: 1.1.1.127) had a higher abundance in feces upon PEC. Subsequently, the abundance of most genes associated with inosine generation from PRPP, such as *purF* (EC: 2.4.2.14), *PFAS* (EC: 6.3.5.3), *purM* (EC: 6.3.3.1), *purE* (EC: 5.4.99.18), *purC* (EC: 6.3.2.6), and *purH* (EC: 2.1.2.3 3.5.4.10), were increased under the condition of PEC. Phylogenetic distribution analysis revealed that the enriched KO genes in the PEC group were manly assigned to Prevotella, Treponema, and Alistipes at the genus level (Fig. 8).

## DISCUSSION

**FIG 9:**
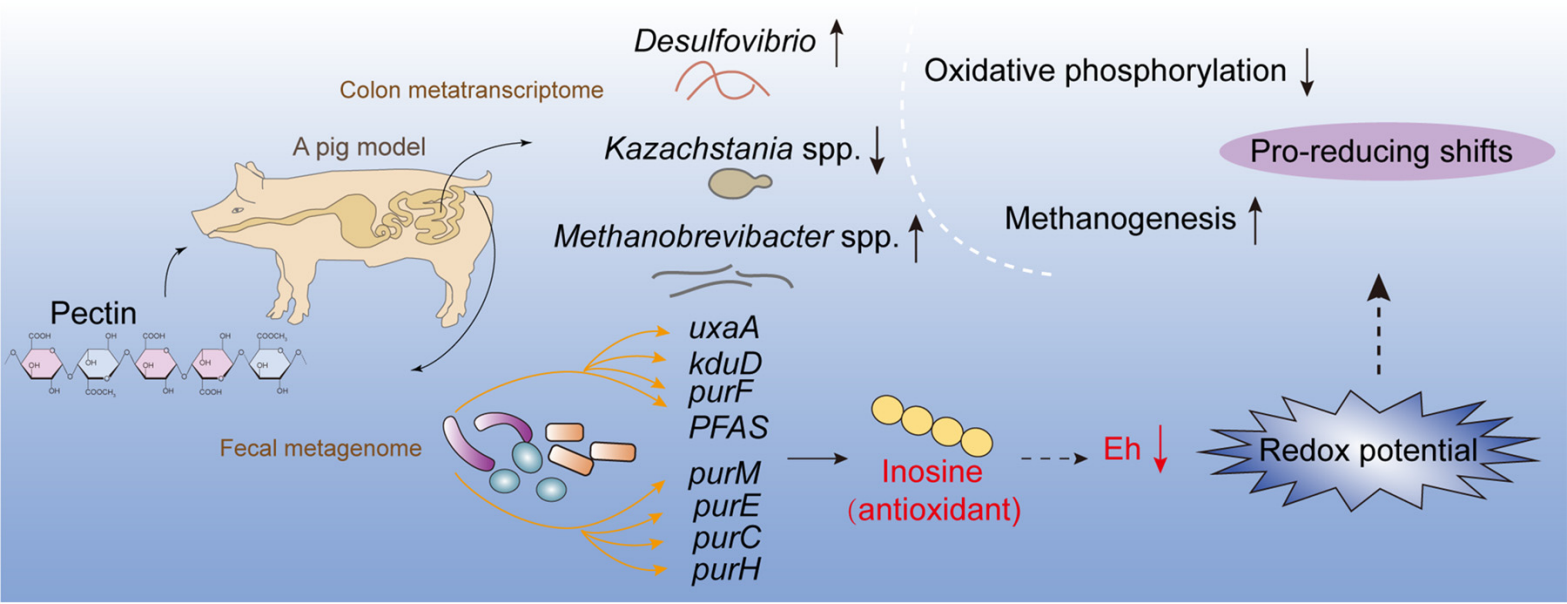
Graphical summary of the main results. Pectin modulates redox-associated microbial taxa (including *Desulfovibrio*, *Kazachstania*, and *Methanobrevibacter*) and functions (oxidative phosphorylation and methanogenesis), which response to changes in redox potential, indicating the proreducing effects of pectin on the redox status of microbial ecosystem. Additionally, hindgut microorganisms generate reducing agents (e.g., inosine) through pectin degradation to decrease the redox potential of microbial ecosystems.

In the world, many factors are threatening intestinal health, such as antibiotics exposure, obesity, and malnutrition. Hence, it is urgent to develop effective therapeutic strategies. Pectin, one of the representative prebiotics that can be used to improve gut health, has a long history as a functional food additive due to its potent health benefits on the gut microbial community. In this study, we first confirmed that pectin could decrease the redox potential in the pig hindgut to modulate microbial composition and functions. Additionally, certain hindgut microorganisms could produce reducing agents (e.g., inosine) through pectin degradation to decrease the redox potential of microbial ecosystems (Fig. 9). Therefore, this study proposes a new mechanism of pectin modulating gut microbial ecosystems and health, and also provides new insights for developing strategies targeting the gut microbiota against diseases.

Generally, bacteria, fungi, and archaea are the cardinal microbial kingdoms that participate in the fermentation of dietary fiber in the large intestine (5, 21, 22). Studies have overwhelmingly showed that the carbohydrate polymer can provide abundant substrates to drive microbial diversity in gut microbiota (5), which was mirrored by the increased bacterial richness after our PEC treatment. Phylogenetic analysis of emerging bacterial species demonstrated that Proteobacteria was the most dominant phylum, which plays a role in helping to maintain the anaerobic environment of the gut for normal microbiome function (10, 23). Besides, *Desulfovibrio* significantly increased in the PEC group, which can take sulfate as the terminal electron acceptor for respiration to produce reducing agents (24), suggesting the enhanced anaerobic environment of gut lumen. As for fungi, the proportion and evenness of fungal community was significantly reduced in the PEC group. Members of the gut microbiome interact directly or indirectly with each other, and cooccurrence network analysis is a tool for a better understanding of these potential interactions (25, 26). Our results showed that keystone taxa in fungi were reduced by PEC and mainly assigned to *Saccharomyces*, *Kluyveromyces*, and *Kazachstania*, which can grow aerobically (27, 28). Functional analysis further revealed that genes involved in microbial oxidative phosphorylation, which is known to generate high-energy ATP for cellular activities (29, 30), occurred predominantly in fungal genus *Kazachstania* and *Saccharomyces* and were massively downregulated by a pectin-enriched diet. The heavy downregulation of genes in the electron transport chain and ATP synthase complexes implied that the reduced generation of ATP might cause insufficient energy supply for cellular metabolic activities (31). Consistent with this, the weakened mRNA surveillance pathway, nucleocytoplasmic transport, and ribosome in fungi at the RNA level indicated that the normal processes (transcription in cell nucleus, transport through nuclear pore complexes, and translation in ribosome) of gene expression in fungal cells was disrupted (32 to 34).

To explore PEC-induced microbial functional differences involved in nutrient metabolism, we analyzed the affected pathways by PEC, including microbial carbon metabolism and amino acid biosynthesis, according to metatranscriptome information. Our results demonstrated that the pentose phosphate pathway was enhanced in PEC pigs, which might be underpinned by the increased pentoses from decarboxylation of pectin (35). This finding suggested the increase of carbohydrate metabolism products, including fructose-6P, glycerate-3P, phosphoenolpyruvate, and pyruvate. Following carbohydrate fermentation, we identified upregulated conversion of pyruvate to methane, suggesting the increased fermentation precursors might improve accessibility of substrate for methanogen to produce methane. Consistently, the finding showed that the relative abundance of Methanobrevibacter smithii, which plays a key role in promoting polysaccharide degradation by removing the H_2_ end product to form methane (36, 37), was higher in the PEC group. In addition, we discovered that amino acid biosynthesis was extensively restrained upon PEC, which was likely related to the decreased functional activities in the fungal community. For example, *Kazachstania* can play an amino acids-providing role in the pig gut (38, 39).

It is known that most fungi generally fit in a microaerobic habitat with relatively high redox potential, and are elevated in some cases such as disease and antibiotic use (40). Thus, depletion of fungi and their oxidative phosphorylation, accompanied by reactive oxygen species generation (41), implied the proreducing state in the colon. Consistently, sulfate-reducing *Desulfovibrio* spp. and methane-producing *Methanobrevibacter* spp., which are representative H_2_-utilizing bacteria (15, 42), increased upon PEC, suggesting more available hydrogen and lower redox potential in the gut lumen of the PEC group. Taken together, these observations demonstrated that pectin-enriched diet induced proreducing shifts on microbial taxa and functions, which changed according to their preferable redox conditions in pig colons, suggesting the potential of pectin to modulate gut microbial ecology.

To further explore whether pectin supplementation can influence the redox state of gut microbial ecology, we performed pilot experiments *in vitro* and *in vivo*, respectively. This observation showed that pectin had no effect on redox potential of *in vitro* cultivation, but significantly decreased that on feces of growing pigs after treatment for 6 days, suggesting the change of pectin on redox potential associated with its metabolic process *in vivo*. The intestinal tract is a complex system with different parameters that vary accordingly to the site *in vivo*, such as oxygen partial pressure, intensity of the dynamic stimuli, and the distribution of microbes, influenced by host conditions (e.g., age, thickness of the mucus layer, and activity of immune cells) (43). Pectin was degraded in such a complex environment in *in vivo* experiments. But in *in vitro* incubation experiments, pectin fermentation was isolated from host conditions in a relatively uniform cultivation medium. These might contribute to no significant difference in redox potential after the inclusion of pectin in an *in vivo* study. Thus, the pH value and redox potential dynamics were determined in fistula pigs upon PEC for 6 days. Notably, only fecal redox potential appears to be decreased by PEC at day 6, and in parallel, there was no other difference between the two groups. This evidence indicated that rather than pH, the changes of redox potential might contribute more to the modulations of microbial communities in the pig hindgut under the condition of PEC, and also suggested that PEC-induced decrease in fecal redox potential might be driven by microorganisms in pectin degradation. This finding was in line with the PEC-induced proreducing shifts in microbial taxa and functions described above and confirmed the proreducing effects of pectin on gut microbial ecology, suggesting that pectin is promising to improve gut redox balance after antibiotics exposure or in the cases of chronic diseases (e.g., obesity, malnutrition, and intestinal damage) (10, 15).

The changes of redox status are generally accompanied by alterations in antioxidants or oxidants in certain environments (20). To search for the potential metabolites responsible for PEC-induced redox potential reduction, an untargeted metabolomics analysis was performed on ileal contents and feces of fistula pigs. We found that upregulated antioxidants were the main contributors to the downward fluctuations of redox potential in feces upon PEC. Among them, inosine, which is an effective antioxidant that can be produced by microorganisms (44, 45), was the most important variable for changes in redox potential, suggesting inosine may be a potent contributor to fecal redox potential decrease under PEC conditions. To trace the source of inosine, we used metagenome sequencing to construct the metabolic routes from pectin degradation pathway to the inosine biosynthesis pathways participated by microorganisms. Interestingly, metagenomic results exhibited that microbiota-derived inosine biosynthesis in the feces was extensively enhanced by PEC, which was in line with the increased inosine described above. This finding also demonstrated that the enrichment of KO enzyme genes involved in pectin degradation occurred predominantly in genus *Prevotella*, which plays an important role in pectin biodegradation (35). Particularly, the KO genes enriched in the conversion from PRPP to inosine were phylogenetically assigned to more diverse genera than those in pectin degradation, suggesting that PRPP as a substrate originated from the pentose phosphate pathway and is broadly available for microorganisms to generate inosine. Altogether, these results manifested that pectin-enriched diet increased the microbiota-derived inosine in feces, which might influence the microbial ecosystem coupled with decreased redox potential. These findings also suggest a new strategy based on redox potential targeting modulation of gut microbial composition to prevent or treat chronic diseases.

### Conclusion.

In summary, our results demonstrated that the abundances of fungal keystone taxa with oxidative phosphorylation were decreased at the RNA level by PEC in pig colons, while those of sulfate-reducing *Desulfovibrio* spp. and methane-producing *Methanobrevibacter* spp. were increased. These proreducing shifts on microbial taxa and functions were consistent with the decreased fecal redox potential upon PEC in a fistula pig model. In addition, a PEC promotes the enrichment of the microbiota-derived antioxidant metabolite inosine, which may induce the redox potential reduction and in turn contribute to PEC-induced modulations of microbial taxa and functions. This investigation proposes a new mechanism of pectin-modulating gut microbial ecosystem and health, and also provides new perspectives for targeting modulation of gut microbiota.

## MATERIALS AND METHODS

### Animals, diets, and experimental design.

This study was carried out according to the guidance of the Animal Care and Use Committee of Nanjing Agricultural University (SYXK [Su] 2015-0656) in China. In experiment 1, a total of 14 pigs (Duroc × Landrace × Large White) with similar body weight (8.8 ± 0.1 kg) were assigned randomly to the control diet (CON) group (*n *= 7, fed with a corn–soybean-based diet) and PEC group (*n *= 7, 8% [wt/wt] of corn starch replaced by pectin in the corn–soybean-based diet). The addition of pectin ranging from 3 to 10% (wt/wt) is reasonable based on literature research (46). The ingredients and nutrient composition of the diets are included in Table S4. Apple pectin (galacturonic acid content = 86.3%, degree of esterification = 10.9%) used in this study was provided by Fufeng Biotechnologies Co. (Xinjiang, China), and more pectin characteristics and corresponding determination methods are shown in Table S5 and the Supplementary Information, respectively. Pigs had free access to feed and water throughout the 40-day experimental period. Finally, all pigs were slaughtered after fasting for 12 h. Luminal contents of proximal colons were collected and stored at −80°C for microbial RNA analysis.

In experiment 2, 12 pigs (Duroc × Landrace × Large White, 16.9 ± 0.2 kg) installed with a fistula in the ileum were randomly distributed into two groups: control group (CON, *n *= 6, fed with a basal diet) and the treated group (PEC, *n *= 6, supplemented with 64 g pectin/kg basal diet). The ingredients of diets are shown in Table S6. The apple pectin was the same as that described in experiment 1. At days 0, 1, 2, 4, and 6, fresh ileal contents and feces were collected immediately for pH and redox potential measurement, and subsequently stored at −80°C for untargeted metabolomic analysis and microbial DNA analysis.

### A pilot experiment *in vivo*.

Before experiment 2, a total of 6 pigs (Duroc × Landrace × Large White) with similar body weight were assigned randomly to the CON group (*n *= 3, fed with a basal diet) and PEC group (*n *= 3, supplemented with 64 g pectin/kg basal diet). The apple pectin was the same as that described in experiment 1. After 6 days, the fecal redox potential of pigs was measured.

### *In vitro* incubations.

Colonic contents used for *in vitro* cultivation were sampled from 3 healthy growing pigs. The details of inocula and basal medium preparation are as in He et al. (47) and Williams et al. (48), respectively. An aliquot (10 mL) of inoculum was subsequently injected into a prewarmed medium containing pectin (10 mg/mL). Negative controls contained all components except pectin. Three bottles were used for each treatment at each sampling time point and incubated at 37°C. Redox potential was measured at 0, 6, 12, 18, and 24 h after inoculation.

### Redox potential measurements.

Redox potentials of ileal contents, fecal samples, and fermentation solutions from *in vitro* cultivations were measured using a ST300/B ORP electrode. All measurements were performed inside an anaerobic chamber under anaerobic conditions.

### RNA extraction, metatranscriptome sequencing and analysis.

Microbial RNA was extracted according to our previous study (6). Eight RNA samples of colon microbiota (four from each group) were randomly selected, followed by sequencing on the Illumina HiSeq X 10 platform. Subsequently, SeqPrep (v.1.1, https://github.com/jstjohn/SeqPrep), Sickle (v.1.33, https://github.com/najoshi/sickle), and BWA (v.0.7.12) (49) were used to trim adaptors and remove low-quality bases, short reads, and host RNA contaminations. Then, Prodigal (v.2.6.3) was used to predict open reading frames (ORFs), and CD-HIT (v.4.6.7) (50) was applied to a construct nonredundant gene catalog with a sequencing identity cutoff of 0.95. Taxonomic assignment and functional annotation of the nonredundant gene catalog were performed according to our previous study (6). Alpha (richness and Shannon index) and beta (Bray-Curtis distance) diversity were calculated using the R vegan package (v.2.5-6) (51). The principal coordinate analysis (PCoA) was performed to show the differences in the microbiota between the two groups, and ANOSIM was calculated using the R vegan package (v.2.5-6) (51). The abundances of genes were calculated in parts per million (PPM) (52). Differentially expressed genes (DEGs) between two different groups were identified according to the PPM.

### Cooccurrence networks.

We calculated the Spearman correlations between microbial species. The networks were constructed according to the positive, significant correlations (*rho* > 0.7 and *P < *0.001) using a Fruchterman-Reingold layout with 10^4^ permutations in igraph. In parallel, we calculated the degrees of cooccurrence (number of direct correlations to a node) of the network.

### Metabolome analysis.

Ileal contents and feces samples were prepared and loaded into an Ultimate 3000LC-Q-Exactive instrument (Thermo, CA, USA) incorporating a Hyper gold C_18_ column (100 mm by 2.1 mm, 1.9 mm; Thermo) to perform a Liquid Chromatograph mass spectrometer (LC-MS) analysis. Procedures of sample preparation and metabolite separation and identification are described in the Supplementary Information. The resulting data matrix was mean-centered and normalized using the sum of the chromatographic peak areas. The fold change (PEC/CON) was calculated based on the normalized data from each group. The sparse PLS discriminant analysis (sPLS-DA) was performed and visualized with 95% confidence interval ellipses (based on the SD) for each group. MetaboAnalyst (v.5.0) was used for metabolic pathway analysis (MetPA).

### DNA extraction, metagenome sequencing.

Microbial DNA was extracted from fecal samples (four samples were randomly selected from each group) using the E.Z.N.A. stool DNA kit (Omega Bio-tek, Norcross, GA, USA) according to manufacturer’s protocols. Metagenomic shotgun sequencing libraries were constructed and sequenced by Shanghai Biozeron Biological Technology Co., Ltd. The raw data were filtered using Trimmomatic (53) and BWA to remove adaptors, low-quality reads, and environmental DNA contamination. Subsequently, the clean reads were assembled by MEGAHIT (v.1.1.1) (54) with a parameter of min-contig-len 500. Then, the contigs with a coverage of less than 60% were removed using Salmon (55). Prodigal was used to predict ORFs (v.2.6.3) (56), and a nonredundant microbial gene set was constructed by CD-HIT (v.4.6.7) (50). Finally, fecal metagenome was constructed and used to analyze the changes in the metagenome function after PEC treatment.

### Statistical analysis.

Values of redox potential and metabolites in ileum contents and feces were compared between CON and PEC pigs using a *t* test (SPSS 21.0). In view of the distribution, the data of metatranscriptome and metagenome were evaluated using a Wilcoxon Mann-Whitney *U* test for group comparison with a significance threshold of *P* < 0.05. In the cooccurrence network, nodes were clustered by fast greedy. Correlations between data sets were calculated using Spearman’s rank correlation.

### Data availability.

The sequencing data of metatranscriptome and metagenome for all samples described above were deposited into the NCBI Sequence Read Archive (SRA) database under accession numbers PRJNA693413 and PRJNA861720, respectively.
