# Supplementary material for: Reduction of Redox Potential Exerts a Key Role in Modulating Gut Microbial Taxa and Function by Dietary Supplementation of Pectin in a Pig Model
Source: Microbiol Spectr. 2022 Dec 8;11(1):e03283-22. doi: 10.1128/spectrum.03283-22 (PMC9927287; doi:10.1128/spectrum.03283-22)
Supplement: Supplemental file 1 — Supplemental material. Download spectrum.03283-22-s0001.pdf, PDF file, 1.2 MB [file spectrum.03283-22-s0001.pdf]

Supplementary Material

Reduction of redox potential exerts a key role in modulating gut microbial taxa and function by dietary supplementation of pectin in a pig model

Rongying Xu, Qiuke Li, Hongyu Wang, Yong Su*, Weiyun Zhu

* **Correspondence:** Corresponding Author: yong.su@njau.edu.cn

Table S1. Effects of pectin-enriched diet on the relative abundance (PPM) of colon bacteria, fungi and archaea at genus level

Genus	CON(PPM)	PEC(PPM)	SEM	*P* Value	Regulated
**Bacteria**					
Unclassified Acidobacteria	0.48	28.77	16.818	0.029	UP
Eggerthella	17.57	52.27	31.51	0.029	UP
Rothia	4.01	0.03	4.185	0.029	DOWN
Trueperella	0.91	0.4	0.332	0.029	DOWN
Corynebacterium	0.2	0.63	0.368	0.029	UP
Kitasatospora	0	0.76	0.625	0.029	UP
Arthrobacter	0.24	0.05	0.124	0.029	DOWN
Yaniella	0.21	0.02	0.154	0.029	DOWN
Anaerophaga	2.52	1377.24	1089.769	0.029	UP
Labilibacter	0.01	843.54	713.003	0.029	UP
Geofilum	5.34	687.91	579.257	0.029	UP
Marinilabilia	0.56	585.3	503.671	0.029	UP
Alkaliflexus	0.45	510.08	438.928	0.029	UP
Thermophagus	0.05	500.76	426.652	0.029	UP
Unclassified Flavobacteriia	2.58	522.55	451.239	0.029	UP
Saccharicrinis	0.01	316.74	276.688	0.029	UP
Alkalitalea	0.05	215.37	177.333	0.029	UP
Petrimonas	18.99	132.38	79.515	0.029	UP
Unclassified Flavobacteriaceae	0	127.32	113.406	0.029	UP
Flavobacterium	22.32	72.65	33.804	0.029	UP
Mariniphaga	0.17	73.75	60.56	0.029	UP
Riemerella	71.43	3.56	56.332	0.029	DOWN
Candidatus Azobacteroides	0	35.35	31.975	0.029	UP
Thermonema	0.09	37.46	29.632	0.029	UP
Williamwhitmania	0.56	33.14	28.444	0.029	UP
Marinifilum	0.18	28.97	23.346	0.021	UP
Unclassified Marinifilaceae	0	28.07	23.252	0.029	UP
Zobellia	0.21	21.38	16.792	0.029	UP
Prolixibacter	0.15	17.96	14.371	0.021	UP
Moheibacter	0	15.18	13.196	0.029	UP
Siphonobacter	0	9.28	12.444	0.029	UP
Salinivirga	0.03	6.09	5.198	0.029	UP
Polaribacter	5.28	0.17	3.639	0.029	DOWN
Flexithrix	0	3.95	3.473	0.029	UP
Reichenbachiella	0.11	4.24	3.726	0.029	UP
Muricauda	0.05	3.37	2.575	0.029	UP
Dyadobacter	0.03	2.84	1.907	0.029	UP
Tangfeifania	0.01	2.74	2.425	0.029	UP
Rufibacter	0.02	2.53	2.406	0.029	UP

Myroides	0.2	1.91	1.138	0.021	UP
Aequorivita	0	1.8	2.092	0.027	UP
Lishizhenia	0	0.98	0.664	0.029	UP
Aureispira	0	0.78	0.682	0.021	UP
Cyclobacterium	0.01	0.31	0.271	0.029	UP
Gilvibacter	0	0.53	0.53	0.029	UP
Psychroserpens	0	0.43	0.378	0.029	UP
Unclassified Candidatus Moranbacteria	0.96	0.1	0.638	0.021	DOWN
Crocosphaera	23.29	0.57	25.538	0.029	DOWN
Phormidium	0.66	0.01	0.38	0.029	DOWN
Aphanizomenon	0.01	0.42	0.432	0.027	UP
Aliterella	0.13	0.03	0.072	0.029	DOWN
Clostridium	23510.96	7731.11	11500.54	0.029	DOWN
Oscillibacter	3382.42	2153.8	797.864	0.029	DOWN
Flavonifractor	2561.56	768.77	1178.972	0.021	DOWN
Anaerofilum	2641.13	52.29	1960.095	0.029	DOWN
Clostridioides	1629.14	287.41	1405.613	0.029	DOWN
Unclassified Peptostreptococcaceae	1488.9	230.36	752.326	0.029	DOWN
Dorea	576.53	355.38	137.616	0.027	DOWN
Terrisporobacter	849.64	51.32	533.562	0.029	DOWN
Pseudoflavonifractor	392.08	143.73	157.396	0.021	DOWN
Angelakisella	249.91	85.27	106.797	0.029	DOWN
Intestinimonas	250.37	78.17	114.56	0.029	DOWN
Hydrogenoanaerobacterium	248.64	13.71	292.227	0.021	DOWN
Negativibacillus	174.71	33.81	102.503	0.021	DOWN
Holdemanella	28.17	160.73	84.994	0.029	UP
Romboutsia	137.99	13.53	89.756	0.027	DOWN
Desnuesiella	63.36	19.93	29.282	0.029	DOWN
Unclassified Oscillospiraceae	46.76	27.39	11.948	0.027	DOWN
Fournierella	56.15	8.71	26.22	0.021	DOWN
Lactococcus	15.34	37.45	14.447	0.029	UP
Provencibacterium	17.42	8.87	6.036	0.029	DOWN
Asaccharospora	21.5	1.22	13.435	0.021	DOWN
Aerococcus	0.51	14.05	11.856	0.027	UP
Papillibacter	11.17	2.27	6.074	0.029	DOWN
Dehalobacter	7.93	3.97	3.132	0.027	DOWN
Peptostreptococcus	8.22	2.09	4.497	0.029	DOWN
Alkaliphilus	5.8	2.33	2.242	0.029	DOWN
Carboxydocella	0	7.27	6.778	0.029	UP
Mogibacterium	0.23	4.43	2.971	0.027	UP
Anaerosphaera	0.04	5.58	7.309	0.029	UP
Tissierella	5.69	0.96	3.207	0.029	DOWN

Sporosarcina	4.67	0.5	3.753	0.029	DOWN
Virgibacillus	3.81	0.4	2.364	0.029	DOWN
Peptococcus	0.4	3.62	2.338	0.029	UP
Unclassified Veillonellaceae	2.41	1.29	0.72	0.029	DOWN
Sporolactobacillus	3.9	0.34	3.225	0.027	DOWN
Caldicellulosiruptor	0.47	2.24	1.063	0.029	UP
Vagococcus	2.52	0.25	1.287	0.029	DOWN
Desulfonispora	0	1.48	1.298	0.021	UP
Murdochiella	1.44	0.1	0.766	0.029	DOWN
Garciella	0.04	1.65	1.644	0.029	UP
Fervidicola	0.01	0.49	0.287	0.029	UP
Jeotgalibacillus	0.6	0.01	0.599	0.021	DOWN
Planomicrobium	0.03	0.28	0.181	0.029	UP
Caldisalinibacter	0.25	0.04	0.144	0.029	DOWN
Pediococcus	0.03	0.16	0.093	0.021	UP
Acetonema	0	0.17	0.149	0.029	UP
Unclassified Paenibacillaceae	0	0.14	0.102	0.029	UP
Cetobacterium	0	0.81	0.703	0.021	UP
Acinetobacter	2984.74	231.48	1985.726	0.029	DOWN
Desulfovibrio	6.39	296.24	222.68	0.021	UP
Rhizobium	0	231.65	327.349	0.029	UP
Halomonas	1.48	47.53	40.539	0.027	UP
Rickettsia	38.19	1.1	27.025	0.029	DOWN
Bradyrhizobium	0	48.75	68.845	0.021	UP
Sphingobium	0.03	32.87	46.172	0.021	UP
Nitrospirillum	0	9.91	7.909	0.021	UP
Methylibium	0.46	7.07	4.879	0.021	UP
Agarivorans	0	5.82	4.94	0.021	UP
Unclassified Succinivibrionaceae	0.31	2.18	1.414	0.021	UP
Pectobacterium	2.1	0.66	0.982	0.021	DOWN
Pigmentiphaga	0.08	2.41	1.625	0.027	UP
Pasteurella	0.04	1.47	1.416	0.027	UP
Unclassified Alteromonadaceae	0	2.35	2.729	0.027	UP
Bordetella	0.4	1.58	0.731	0.027	UP
Pantoea	0.26	1.56	0.848	0.027	UP
Turicimonas	0.14	1.23	0.731	0.029	UP
Xenorhabdus	1.13	0.18	0.718	0.021	DOWN
Desulfomicrobium	0	1.26	1.244	0.027	UP
Magnetospira	0.02	1.05	0.812	0.021	UP
Unclassified Xanthomonadaceae	0	0.92	0.775	0.029	UP
Desulfarculus	0	0.72	0.531	0.029	UP

Candidatus Dactylopiibacterium	0	0.7	0.588	0.029	UP
Methylophaga	0.07	0.56	0.313	0.029	UP
Azohydromonas	0.57	0.07	0.318	0.029	DOWN
Natronohydrobacter	0	0.16	0.123	0.021	UP
Insolitispirillum	0	0.16	0.103	0.021	UP
Roseospirillum	0.12	0.01	0.106	0.021	DOWN
Robbsia	0	0.1	0.061	0.029	UP
Mycoplasma	43.77	5.02	27.432	0.021	DOWN
Unclassified Verrucomicrobiales	0.03	8.62	7.625	0.027	UP
Unclassified Verrucomicrobiae	0	0.95	0.681	0.021	UP
**Fungi**					
Kazachstania	4708.77	48.89	1857.147	0.029	DOWN
Nakaseomyces	1121.53	22.47	304.98	0.029	DOWN
Batrachochytrium	616.1	95.1	112.631	0.029	DOWN
Saccharomyces	1265.84	7.41	509.354	0.029	DOWN
Kluyveromyces	883.22	4.01	367.445	0.029	DOWN
Spathaspora	599.89	11.35	236.148	0.029	DOWN
Naumovozyma	666.31	3.86	263.075	0.029	DOWN
Tetrapisispora	615.78	1.84	257.809	0.029	DOWN
Penicillium	598.77	6.77	265.937	0.029	DOWN
Lachancea	248.84	10.6	87.124	0.029	DOWN
Vanderwaltozyma	313.93	1.22	123.153	0.029	DOWN
Eremothecium	127.78	0.66	52.869	0.029	DOWN
Torulaspora	54.92	0.44	19.992	0.029	DOWN
Hanseniaspora	67.88	0.17	28.651	0.029	DOWN
Komagataella	22.7	0	8.563	0.029	DOWN
Talaromyces	22.2	0.1	8.683	0.029	DOWN
Ophiostoma	2.62	0.3	0.645	0.029	DOWN
Cyberlindnera	3.92	0.15	1.458	0.029	DOWN
Anaeromyces	0.9	15.15	3.507	0.029	UP
Rasamsonia	1.52	0	0.479	0.029	DOWN
Arthrobotrys	0.79	0.08	0.186	0.029	DOWN
Pyronema	0.62	0.02	0.15	0.029	DOWN
Verticillium	0.41	0.04	0.106	0.029	DOWN
Setosphaeria	0.15	0.01	0.038	0.029	DOWN
Coccidioides	0.1	0.02	0.022	0.029	DOWN
**Archaea**					
Methanobrevibacter	3.16	133.96	56.187	0.029	UP
Methanocorpusculum	0.77	0.13	0.137	0.029	DOWN

Table S2. Composition of metabolic pathways based on the first-level and second-level functions in the KEGG

ko	Definition	First Level KEGG function	Second Level KEGG function	Mean (parts per million)
ko02030	Bacterial chemotaxis	Cellular Processes	Cell motility	513.299
ko02040	Flagellar assembly	Cellular Processes	Cell motility	549.915
ko04110	Cell cycle	Cellular Processes	Cell growth and death	52.854
ko04111	Cell cycle - yeast	Cellular Processes	Cell growth and death	25.73
ko04112	Cell cycle - Caulobacter	Cellular Processes	Cell growth and death	695.595
ko04113	Meiosis - yeast	Cellular Processes	Cell growth and death	29.671
ko04114	Oocyte meiosis	Cellular Processes	Cell growth and death	85.61
ko04115	p53 signaling pathway	Cellular Processes	Cell growth and death	49.318
ko04139	Regulation of mitophagy - yeast	Cellular Processes	Transport and catabolism	12.765
ko04140	Regulation of autophagy	Cellular Processes	Transport and catabolism	2.728
ko04142	Lysosome	Cellular Processes	Transport and catabolism	245.305
ko04144	Endocytosis	Cellular Processes	Transport and catabolism	895.233
ko04145	Phagosome	Cellular Processes	Transport and catabolism	314.708
ko04146	Peroxisome	Cellular Processes	Transport and catabolism	388.593
ko04210	Apoptosis	Cellular Processes	Cell growth and death	196.588
ko04214	Apoptosis - fly	Cellular Processes	Cell growth and death	115.316
ko04215	Apoptosis - multiple species	Cellular Processes	Cell growth and death	45.886
ko04510	Focal adhesion	Cellular Processes	Cellular community	80.926
ko04520	Adherens junction	Cellular Processes	Cellular community	59.147
ko04530	Tight junction	Cellular Processes	Cellular community	72.458
ko04540	Gap junction	Cellular Processes	Cellular community	173.736
ko04550	Signaling pathways regulating pluripotency of stem cells	Cellular Processes	Cellular community	18.25
ko04810	Regulation of actin cytoskeleton	Cellular Processes	Cell motility	91.713

ko02010	ABC transporters	Environmental Information Processing	Membrane transport	2261.625
ko02020	Two-component system	Environmental Information Processing	Signal transduction	1515.619
ko02060	Phosphotransferase system (PTS)	Environmental Information Processing	Membrane transport	354.412
ko03070	Bacterial secretion system	Environmental Information Processing	Membrane transport	2629.723
ko04010	MAPK signaling pathway	Environmental Information Processing	Signal transduction	91.593
ko04011	MAPK signaling pathway - yeast	Environmental Information Processing	Signal transduction	76.061
ko04012	ErbB signaling pathway	Environmental Information Processing	Signal transduction	20.041
ko04013	MAPK signaling pathway - fly	Environmental Information Processing	Signal transduction	11.523
ko04014	Ras signaling pathway	Environmental Information Processing	Signal transduction	85.002
ko04015	Rap1 signaling pathway	Environmental Information Processing	Signal transduction	105.126
ko04020	Calcium signaling pathway	Environmental Information Processing	Signal transduction	64.066
ko04022	cGMP-PKG signaling pathway	Environmental Information Processing	Signal transduction	72.451
ko04024	cAMP signaling pathway	Environmental Information Processing	Signal transduction	68.537
ko04060	Cytokine-cytokine receptor interaction	Environmental Information Processing	Signaling molecules and interaction	0.508

ko04064	NF-kappa B signaling pathway	Environmental Information Processing	Signal transduction	6.522
ko04066	HIF-1 signaling pathway	Environmental Information Processing	Signal transduction	2014.058
ko04068	FoxO signaling pathway	Environmental Information Processing	Signal transduction	177.162
ko04070	Phosphatidylinositol signaling system	Environmental Information Processing	Signal transduction	96.01
ko04071	Sphingolipid signaling pathway	Environmental Information Processing	Signal transduction	41.737
ko04072	Phospholipase D signaling pathway	Environmental Information Processing	Signal transduction	106.928
ko04080	Neuroactive ligand-receptor interaction	Environmental Information Processing	Signaling molecules and interaction	0.34
ko04150	mTOR signaling pathway	Environmental Information Processing	Signal transduction	151.838
ko04151	PI3K-Akt signaling pathway	Environmental Information Processing	Signal transduction	359.577
ko04152	AMPK signaling pathway	Environmental Information Processing	Signal transduction	389.002
ko04310	Wnt signaling pathway	Environmental Information Processing	Signal transduction	33.703
ko04330	Notch signaling pathway	Environmental Information Processing	Signal transduction	3.553
ko04340	Hedgehog signaling pathway	Environmental Information Processing	Signal transduction	4.153
ko04350	TGF-beta signaling pathway	Environmental Information Processing	Signal transduction	13.71

ko04370	VEGF signaling pathway	Environmental Information Processing	Signal transduction	33.119
ko04390	Hippo signaling pathway	Environmental Information Processing	Signal transduction	86.17
ko04391	Hippo signaling pathway - fly	Environmental Information Processing	Signal transduction	79.93
ko04512	ECM-receptor interaction	Environmental Information Processing	Signaling molecules and interaction	2.986
ko04514	Cell adhesion molecules (CAMs)	Environmental Information Processing	Signaling molecules and interaction	5.411
ko04630	Jak-STAT signaling pathway	Environmental Information Processing	Signal transduction	8.106
ko04668	TNF signaling pathway	Environmental Information Processing	Signal transduction	9.05
ko00970	Aminoacyl-tRNA biosynthesis	Genetic Information Processing	Translation	2260.985
ko03008	Ribosome biogenesis in eukaryotes	Genetic Information Processing	Translation	143.712
ko03010	Ribosome	Genetic Information Processing	Translation	44768.891
ko03013	RNA transport	Genetic Information Processing	Translation	517.719
ko03015	mRNA surveillance pathway	Genetic Information Processing	Translation	35.805
ko03018	RNA degradation	Genetic Information Processing	Folding, sorting and degradation	2502.075
ko03020	RNA polymerase	Genetic Information Processing	Transcription	999.387
ko03022	Basal transcription factors	Genetic Information Processing	Transcription	12.042
ko03030	DNA replication	Genetic Information Processing	Replication and repair	928.94

ko03040	Spliceosome	Genetic Information Processing	Transcription	84.006
ko03050	Proteasome	Genetic Information Processing	Folding, sorting and degradation	51.994
ko03060	Protein export	Genetic Information Processing	Folding, sorting and degradation	2775.2
ko03410	Base excision repair	Genetic Information Processing	Replication and repair	317.587
ko03420	Nucleotide excision repair	Genetic Information Processing	Replication and repair	212.859
ko03430	Mismatch repair	Genetic Information Processing	Replication and repair	1004.884
ko03440	Homologous recombination	Genetic Information Processing	Replication and repair	1244.361
ko03450	Non-homologous end-joining	Genetic Information Processing	Replication and repair	7.452
ko03460	Fanconi anemia pathway	Genetic Information Processing	Replication and repair	3.121
ko04120	Ubiquitin mediated proteolysis	Genetic Information Processing	Folding, sorting and degradation	21.672
ko04122	Sulfur relay system	Genetic Information Processing	Folding, sorting and degradation	205.991
ko04130	SNARE interactions in vesicular transport	Genetic Information Processing	Folding, sorting and degradation	3.98
ko04141	Protein processing in endoplasmic reticulum	Genetic Information Processing	Folding, sorting and degradation	637.701
ko00010	Glycolysis / Gluconeogenesis	Metabolism	Carbohydrate metabolism	6531.309
ko00020	Citrate cycle (TCA cycle)	Metabolism	Carbohydrate metabolism	3037.276
ko00030	Pentose phosphate pathway	Metabolism	Carbohydrate metabolism	2560.387
ko00040	Pentose and glucuronate	Metabolism	Carbohydrate metabolism	1050.157

	interconversions			
ko00051	Fructose and mannose metabolism	Metabolism	Carbohydrate metabolism	2896.522
ko00052	Galactose metabolism	Metabolism	Carbohydrate metabolism	2276.893
ko00053	Ascorbate and aldarate metabolism	Metabolism	Carbohydrate metabolism	171.847
ko00061	Fatty acid biosynthesis	Metabolism	Lipid metabolism	1202.36
ko00062	Fatty acid elongation	Metabolism	Lipid metabolism	3.102
ko00071	Fatty acid degradation	Metabolism	Lipid metabolism	679.145
ko00072	Synthesis and degradation of ketone bodies	Metabolism	Lipid metabolism	109.411
ko00100	Steroid biosynthesis	Metabolism	Lipid metabolism	3.877
ko00120	Primary bile acid biosynthesis	Metabolism	Lipid metabolism	101.145
ko00121	Secondary bile acid biosynthesis	Metabolism	Lipid metabolism	99.385
ko00130	Ubiquinone and other terpenoid-quinone biosynthesis	Metabolism	Metabolism of cofactors and vitamins	120.006
ko00140	Steroid hormone biosynthesis	Metabolism	Lipid metabolism	12.031
ko00190	Oxidative phosphorylation	Metabolism	Energy metabolism	5079.924
ko00195	Photosynthesis	Metabolism	Energy metabolism	2755.636
ko00220	Arginine biosynthesis	Metabolism	Amino acid metabolism	1365.899
ko00230	Purine metabolism	Metabolism	Nucleotide metabolism	5503.422

ko00232	Caffeine metabolism	Metabolism	Biosynthesis of other secondary metabolites	1.085
ko00240	Pyrimidine metabolism	Metabolism	Nucleotide metabolism	4634.703
ko00250	Alanine, aspartate and glutamate metabolism	Metabolism	Amino acid metabolism	2285.598
ko00253	Tetracycline biosynthesis	Metabolism	Metabolism of terpenoids and polyketides	41.973
ko00254	Aflatoxin biosynthesis	Metabolism	Biosynthesis of other secondary metabolites	0.222
ko00260	Glycine, serine and threonine metabolism	Metabolism	Amino acid metabolism	2256.45
ko00261	Monobactam biosynthesis	Metabolism	Biosynthesis of other secondary metabolites	378.011
ko00270	Cysteine and methionine metabolism	Metabolism	Amino acid metabolism	1746.781
ko00280	Valine, leucine and isoleucine degradation	Metabolism	Amino acid metabolism	1114.375
ko00281	Geraniol degradation	Metabolism	Metabolism of terpenoids and polyketides	84.327
ko00290	Valine, leucine and isoleucine biosynthesis	Metabolism	Amino acid metabolism	901.857
ko00300	Lysine biosynthesis	Metabolism	Amino acid metabolism	836.631
ko00310	Lysine degradation	Metabolism	Amino acid metabolism	321.009
ko00311	Penicillin and cephalosporin biosynthesis	Metabolism	Biosynthesis of other secondary metabolites	39.401
ko00330	Arginine and proline metabolism	Metabolism	Amino acid metabolism	544.098
ko00332	Carbapenem biosynthesis	Metabolism	Biosynthesis of other secondary metabolites	56.354
ko00340	Histidine metabolism	Metabolism	Amino acid metabolism	488.191

ko00350	Tyrosine metabolism	Metabolism	Amino acid metabolism	556.611
ko00360	Phenylalanine metabolism	Metabolism	Amino acid metabolism	441.192
ko00361	Chlorocyclohexane and chlorobenzene degradation	Metabolism	Xenobiotics biodegradation and metabolism	21.791
ko00362	Benzoate degradation	Metabolism	Xenobiotics biodegradation and metabolism	271.927
ko00364	Fluorobenzoate degradation	Metabolism	Xenobiotics biodegradation and metabolism	10.503
ko00380	Tryptophan metabolism	Metabolism	Amino acid metabolism	208.776
ko00400	Phenylalanine, tyrosine and tryptophan biosynthesis	Metabolism	Amino acid metabolism	725.302
ko00401	Novobiocin biosynthesis	Metabolism	Biosynthesis of other secondary metabolites	225.806
ko00410	beta-Alanine metabolism	Metabolism	Metabolism of other amino acids	219.643
ko00430	Taurine and hypotaurine metabolism	Metabolism	Metabolism of other amino acids	509.255
ko00440	Phosphonate and phosphinate metabolism	Metabolism	Metabolism of other amino acids	51.329
ko00450	Selenocompound metabolism	Metabolism	Metabolism of other amino acids	446.006
ko00460	Cyanoamino acid metabolism	Metabolism	Metabolism of other amino acids	432.822
ko00471	D-Glutamine and D-glutamate metabolism	Metabolism	Metabolism of other amino acids	136.839
ko00472	D-Arginine and D-ornithine metabolism	Metabolism	Metabolism of other amino acids	5.879

ko00473	D-Alanine metabolism	Metabolism	Metabolism of other amino acids	83.648
ko00480	Glutathione metabolism	Metabolism	Metabolism of other amino acids	423.11
ko00500	Starch and sucrose metabolism	Metabolism	Carbohydrate metabolism	2408.148
ko00510	N-Glycan biosynthesis	Metabolism	Glycan biosynthesis and metabolism	83.508
ko00511	Other glycan degradation	Metabolism	Glycan biosynthesis and metabolism	770.875
ko00512	Mucin type O-Glycan biosynthesis	Metabolism	Glycan biosynthesis and metabolism	0.116
ko00513	Various types of N-glycan biosynthesis	Metabolism	Glycan biosynthesis and metabolism	4.105
ko00514	Other types of O-glycan biosynthesis	Metabolism	Glycan biosynthesis and metabolism	2.486
ko00520	Amino sugar and nucleotide sugar metabolism	Metabolism	Carbohydrate metabolism	3575.195
ko00521	Streptomycin biosynthesis	Metabolism	Biosynthesis of other secondary metabolites	1327.127
ko00523	Polyketide sugar unit biosynthesis	Metabolism	Metabolism of terpenoids and polyketides	424.846
ko00524	Butirosin and neomycin biosynthesis	Metabolism	Biosynthesis of other secondary metabolites	421.18
ko00531	Glycosaminoglycan degradation	Metabolism	Glycan biosynthesis and metabolism	139.773
ko00540	Lipopolysaccharide biosynthesis	Metabolism	Glycan biosynthesis and metabolism	617.746
ko00550	Peptidoglycan biosynthesis	Metabolism	Glycan biosynthesis and metabolism	580.247
ko00561	Glycerolipid metabolism	Metabolism	Lipid metabolism	258.888

ko00562	Inositol phosphate metabolism	Metabolism	Carbohydrate metabolism	597.996
ko00563	Glycosylphosphatidylinositol(GPI)-anchor biosynthesis	Metabolism	Glycan biosynthesis and metabolism	3.609
ko00564	Glycerophospholipid metabolism	Metabolism	Lipid metabolism	462.563
ko00565	Ether lipid metabolism	Metabolism	Lipid metabolism	3.563
ko00590	Arachidonic acid metabolism	Metabolism	Lipid metabolism	48.213
ko00591	Linoleic acid metabolism	Metabolism	Lipid metabolism	1.839
ko00592	alpha-Linolenic acid metabolism	Metabolism	Lipid metabolism	7.45
ko00600	Sphingolipid metabolism	Metabolism	Lipid metabolism	246.358
ko00601	Glycosphingolipid biosynthesis - lacto and neolacto series	Metabolism	Glycan biosynthesis and metabolism	0.067
ko00603	Glycosphingolipid biosynthesis - globo series	Metabolism	Glycan biosynthesis and metabolism	193.8
ko00604	Glycosphingolipid biosynthesis - ganglio series	Metabolism	Glycan biosynthesis and metabolism	115.32
ko00620	Pyruvate metabolism	Metabolism	Carbohydrate metabolism	3652.573
ko00621	Dioxin degradation	Metabolism	Xenobiotics biodegradation and metabolism	21.603
ko00622	Xylene degradation	Metabolism	Xenobiotics biodegradation and metabolism	23.992

ko00623	Toluene degradation	Metabolism	Xenobiotics biodegradation and metabolism	8.001
ko00624	Polycyclic aromatic hydrocarbon degradation	Metabolism	Xenobiotics biodegradation and metabolism	4.697
ko00625	Chloroalkane and chloroalkene degradation	Metabolism	Xenobiotics biodegradation and metabolism	324.888
ko00626	Naphthalene degradation	Metabolism	Xenobiotics biodegradation and metabolism	277.16
ko00627	Aminobenzoate degradation	Metabolism	Xenobiotics biodegradation and metabolism	110.421
ko00630	Glyoxylate and dicarboxylate metabolism	Metabolism	Carbohydrate metabolism	2712.359
ko00633	Nitrotoluene degradation	Metabolism	Xenobiotics biodegradation and metabolism	158.468
ko00640	Propanoate metabolism	Metabolism	Carbohydrate metabolism	1690.843
ko00642	Ethylbenzene degradation	Metabolism	Xenobiotics biodegradation and metabolism	8.78
ko00643	Styrene degradation	Metabolism	Xenobiotics biodegradation and metabolism	71.453
ko00650	Butanoate metabolism	Metabolism	Carbohydrate metabolism	2286.213
ko00660	C5-Branched dibasic acid metabolism	Metabolism	Carbohydrate metabolism	425.293
ko00670	One carbon pool by folate	Metabolism	Metabolism of cofactors and vitamins	1124.432
ko00680	Methane metabolism	Metabolism	Energy metabolism	3375.578
ko00710	Carbon fixation in photosynthetic organisms	Metabolism	Energy metabolism	5037.767

ko00720	Carbon fixation pathways in prokaryotes	Metabolism	Energy metabolism	4468.64
ko00730	Thiamine metabolism	Metabolism	Metabolism of cofactors and vitamins	347.957
ko00740	Riboflavin metabolism	Metabolism	Metabolism of cofactors and vitamins	326.842
ko00750	Vitamin B6 metabolism	Metabolism	Metabolism of cofactors and vitamins	391.354
ko00760	Nicotinate and nicotinamide metabolism	Metabolism	Metabolism of cofactors and vitamins	669.955
ko00770	Pantothenate and CoA biosynthesis	Metabolism	Metabolism of cofactors and vitamins	995.48
ko00780	Biotin metabolism	Metabolism	Metabolism of cofactors and vitamins	685.278
ko00785	Lipoic acid metabolism	Metabolism	Metabolism of cofactors and vitamins	50.292
ko00790	Folate biosynthesis	Metabolism	Metabolism of cofactors and vitamins	782.445
ko00791	Atrazine degradation	Metabolism	Xenobiotics biodegradation and metabolism	59.468
ko00830	Retinol metabolism	Metabolism	Metabolism of cofactors and vitamins	46.006
ko00860	Porphyrin and chlorophyll metabolism	Metabolism	Metabolism of cofactors and vitamins	322.933
ko00900	Terpenoid backbone biosynthesis	Metabolism	Metabolism of terpenoids and polyketides	869.706
ko00901	Indole alkaloid biosynthesis	Metabolism	Biosynthesis of other secondary metabolites	1.706
ko00903	Limonene and pinene degradation	Metabolism	Metabolism of terpenoids and polyketides	36.243
ko00906	Carotenoid biosynthesis	Metabolism	Metabolism of terpenoids and polyketides	2.099
ko00908	Zeatin biosynthesis	Metabolism	Metabolism of terpenoids and polyketides	164.643

ko00909	Sesquiterpenoid and triterpenoid biosynthesis	Metabolism	Metabolism of terpenoids and polyketides	0.426
ko00910	Nitrogen metabolism	Metabolism	Energy metabolism	1017.992
ko00920	Sulfur metabolism	Metabolism	Energy metabolism	373.672
ko00930	Caprolactam degradation	Metabolism	Xenobiotics biodegradation and metabolism	27.638
ko00940	Phenylpropanoid biosynthesis	Metabolism	Biosynthesis of other secondary metabolites	167.733
ko00944	Flavone and flavonol biosynthesis	Metabolism	Biosynthesis of other secondary metabolites	0.384
ko00950	Isoquinoline alkaloid biosynthesis	Metabolism	Biosynthesis of other secondary metabolites	196.502
ko00960	Tropane, piperidine and pyridine alkaloid biosynthesis	Metabolism	Biosynthesis of other secondary metabolites	235.695
ko00965	Betalain biosynthesis	Metabolism	Biosynthesis of other secondary metabolites	1.853
ko00980	Metabolism of xenobiotics by cytochrome P450	Metabolism	Xenobiotics biodegradation and metabolism	80.454
ko00982	Drug metabolism - cytochrome P450	Metabolism	Xenobiotics biodegradation and metabolism	86.04
ko00983	Drug metabolism - other enzymes	Metabolism	Xenobiotics biodegradation and metabolism	603.102
ko01040	Biosynthesis of unsaturated fatty acids	Metabolism	Lipid metabolism	283.542
ko01051	Biosynthesis of ansamycins	Metabolism	Metabolism of terpenoids and polyketides	238.222

ko01053	Biosynthesis of siderophore group nonribosomal peptides	Metabolism	Metabolism of terpenoids and polyketides	4.663
ko01054	Nonribosomal peptide structures	Metabolism	Metabolism of terpenoids and polyketides	6.058
ko01055	Biosynthesis of vancomycin group antibiotics	Metabolism	Metabolism of terpenoids and polyketides	121.117
ko01100	Metabolic pathways	Metabolism	Global and overview maps	41751.572
ko01110	Biosynthesis of secondary metabolites	Metabolism	Global and overview maps	18265.59
ko01120	Microbial metabolism in diverse environments	Metabolism	Global and overview maps	15404.362
ko01130	Biosynthesis of antibiotics	Metabolism	Global and overview maps	15870.332
ko01200	Carbon metabolism	Metabolism	Global and overview maps	12588.702
ko01210	2-Oxocarboxylic acid metabolism	Metabolism	Global and overview maps	1610.122
ko01212	Fatty acid metabolism	Metabolism	Global and overview maps	1423.851
ko01220	Degradation of aromatic compounds	Metabolism	Global and overview maps	358.586
ko01230	Biosynthesis of amino acids	Metabolism	Global and overview maps	9555.564

Table S3. Differential ileal and faecal metabolites between the CON and PEC groups

Compartments	Metabolites	CON (relative peak area)	PEC (relative peak area)	SEM	Fold change	*P* Value
Ileum	Neohesperidin	0.3	306.38	57.046	1022.44	0.001
	Neodiosmin	0.14	51.16	8.76	370.53	0.002
	18-β-Glycyrrhetinic acid	1.04	8.65	1.346	8.28	0.003
	Farrerol	0.39	2.28	0.396	5.92	0.02
	Resolvin E1	4.42	18.63	3.416	4.21	0.049
	Nicotinamide	16.38	45.98	6.421	2.81	0.024
	Pentadecanoyl Ethanolamide	1.12	3	0.438	2.68	0.037
	3-Hydroxypicolinic acid	23.5	41.85	4.67	1.78	0.042
	Ala-Phe	66.77	48.33	4.582	0.72	0.048
	Asiaticoside	16.18	9.95	1.61	0.61	0.046
	hydroquinone	22.99	13.87	2.111	0.6	0.022
	7,8-dimethylalloxazine (lumichrome)	2.97	1.78	0.292	0.6	0.033
	Quinoline-2-carboxylic acid	3.02	1.72	0.326	0.57	0.038
	Atenolol-desisopropyl	3.73	2.01	0.431	0.54	0.038
	naringenin-7-O-glucoside	35.18	18.79	3.718	0.53	0.028
	Agmatine	125.79	61.79	15.856	0.49	0.036
	Genistin	209.7	98.7	27.828	0.47	0.039
	Cholest-4,6-Dien-3-One	197.37	81.58	25.903	0.41	0.029
	Genistein	396.03	156.34	44.914	0.39	0.002
	(S)-Equol	17.37	6.48	2.438	0.37	0.016
	Phenylacetylglycine	85.13	29.79	13.668	0.35	0.035
	afzelin	54.64	11.37	10.106	0.21	0.039
	quercitrin	149.43	16.66	26.951	0.11	0.016
	apigenin-7-O-glucuronide	57.42	5.92	9.879	0.1	0.002
	(R)-Equol	13.85	0.85	2.768	0.06	0.024
	oroxindin	231	9.77	39.469	0.04	0.004
	Baicalin	2739.75	107.17	539.85	0.04	0.019
Feces	Aconitic Acid	8.76	30.26	4.036	3.45	0.002

Phenylpyruvic acid	271.43	763.44	111.487	2.81	0.018
Inosine	57.07	153.54	25.08	2.69	0.048
D-GLUCURONIC ACID	693.45	1817.49	237.561	2.62	0.009
Phosphatidylethanolamine lyso 18:0	241.82	569.09	75.583	2.35	0.033
Phosphatidylethanolamine lyso 16:0	1631.73	3432.41	386.756	2.1	0.011
Ascorbic acid	624.8	1239.88	157.758	1.98	0.044
2-Oxoglutaric acid	1215.8	2377.53	279.495	1.96	0.029
trans-Aconitic acid	3.01	5.78	0.637	1.92	0.021
1-Stearoyl-sn-glycero-3-phosphocholine	296.54	552.76	66.804	1.86	0.049
3-Hydroxypicolinic acid	68.31	125.74	13.591	1.84	0.026
Creatine	1586.66	2900.45	268.175	1.83	0.006
Valine	330.49	586.27	56.756	1.77	0.015
L-Valine	394.11	693.72	52.933	1.76	0
N-α-L-Acetyl-arginine	27.96	46.35	4.355	1.66	0.026
Hypoxanthine	2219.14	3590.63	273.094	1.62	0.004
Isophorone	123.6	197.94	19.08	1.6	0.045
Thymine	962.41	1449.68	117.472	1.51	0.03
Indole-3-acetic acid	18.8	28.28	2.333	1.5	0.034
1-Methylguanine	28.69	42.11	2.545	1.47	0.002
4-Hydroxybenzaldehyde	171.87	251.01	16.674	1.46	0.009
7-Methylguanine	31.41	45.24	2.58	1.44	0.001
N-Acetylmuramic Acid	376.11	530.03	36.887	1.41	0.028
Uracil	326.8	460.26	32.45	1.41	0.032
Caprylic acid	82.8	115.79	8.176	1.4	0.036
Pantothenic acid	423.98	589.49	40.531	1.39	0.033
L-Norleucine	5740.13	7962.85	569.301	1.39	0.044
Dioctyl Phthalate	14.86	19.77	1.212	1.33	0.035
Cinnamoylglycine	1.19	1.57	0.09	1.31	0.028
L-Lysine	815.94	1069.59	64.24	1.31	0.041
Di-n-butyl phthalate	165.21	200.42	8.278	1.21	0.025
DEET	31.06	36.07	1.09	1.16	0.013
Crotamiton	298.85	338.85	9.506	1.13	0.027

Oxycodone	67.49	55.14	3.187	0.82	0.046
Prespatane	81.46	59.47	5.012	0.73	0.019
3-Hydroxy-3-methylglutarate	67.83	46.44	5.261	0.68	0.034
Phacidin	20.15	11.62	1.814	0.58	0.01
Deoxycholic acid	38480.35	16164.36	5635.94	0.42	0.04
Indole-3-carbinol	322.1	109.9	50.706	0.34	0.028
Sulfocholic acid	60.64	19.98	10.538	0.33	0.047

Table S4. Composition and analyzed nutrient contents of experimental diets (as-fed basis) in experiment 1

Diet	CON	PEC
Ingredients (g/kg)		
Corn starch	80	0
Inulin	0	0
Raw potato starch	0	0
Pectin	0	80
Corn	518	518
Wheat bran	90	90
Soybean meal	260	260
Fish meal	20	20
Dicalcium phosphate	9	9
Limestone	9	9
Salt	3	3
Vitamin and mineral premix[a]	10	10
L-Lysine	1	1
Nutrient analysis (g/kg)		
CP	194.3	194.5
Ash	79.7	79.7
EE	21.2	21.1

a This mineral and vitamin premix (1%) supplies per kg diet as follows: VA 11 000 IU, VD3 1 000 IU, VE 16 IU, VK1 1mg, VB1 0.6 mg, VB2 0.6 mg, d-pantothenic acid 6 mg, nicotinic acid 10 mg, VB12 0.03 mg, folic acid 0.8 mg, VB6 1.5 mg, choline 800 mg, Fe 165 mg, Zn 165 mg, Cu 16.5 mg, Mn 30 mg, Co 0.15 mg, I 0.25 mg, Se 0.25 mg. CON, a control diet;PEC, a pectin enriched diet.

Table S5. Physico-chemical characterization of apple pectin

Items	Values
Loss on drying (%)	10.40
Ash (%)	7.6
Acid infusible ash	0.1
SO_2 (mg/kg)	9.9
Trace ethanol (%)	0.043
Pb (mg/kg)	≤2
DM (%)	10.9
GalA content (%)	86.3

Table S6. Composition and analyzed nutrient contents of basal diet in experiment 2

Items	Basal diet
Ingredients (%)	
Corn	72.2
Soybean meal	24
Soybean oil	0.37
Dicalcium phosphate	0.95
Limestone	0.95
Salt	0.3
L-Lysine, 78%	0.32
DL-Methionine	0.06
L-Threonine	0.08
L-Tryptophan	0.02
Vitamin and mineral premix[a]	10
Chromium oxide	0.25
Nutrient analysis (g/kg)	
DM	87.53
CP	17.37
Ash	5.46

a This mineral and vitamin premix (1%) supplies per kg diet as follows: VA 6 000 IU, VD3 1 700 IU, VE 27 IU, VK3 2.5 mg, VB1 1.5 mg, VB2 6 mg, VB6 2.5 mg, VB12 30 μg, pantothenic acid 17.5 mg, nicotinic acid 25 mg, folic acid 2.5 mg, biotin 0.6 mg, choline chloride 300 mg, Fe 108 mg, Zn 75 mg, Cu 15 mg, Mn 46 mg, I 1.2 mg, Se 0.3 mg.

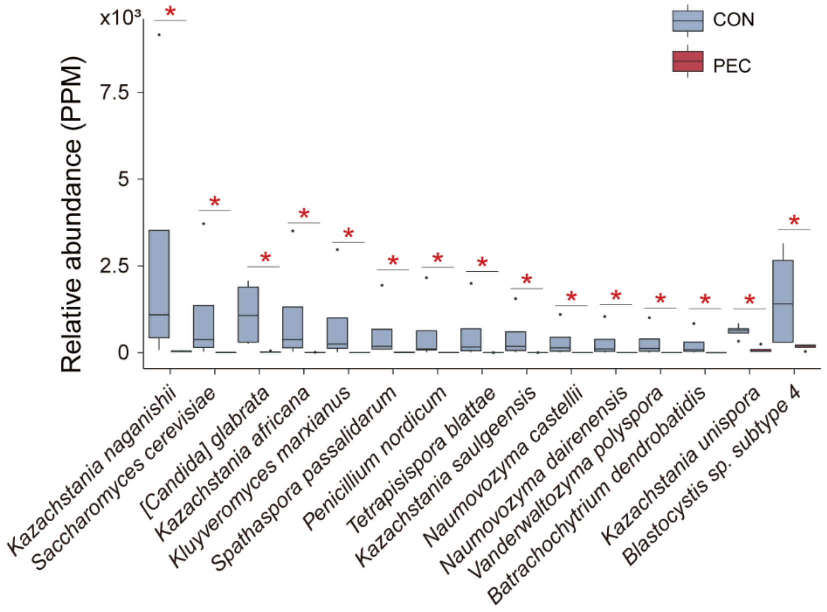

FIG S1 Differential fungal species that more than 0.5% at least one group were compared between the CON and PEC groups. Significantly different species were tested by Mann-Whiney *U* test with *P* value of < 0.05 (*).

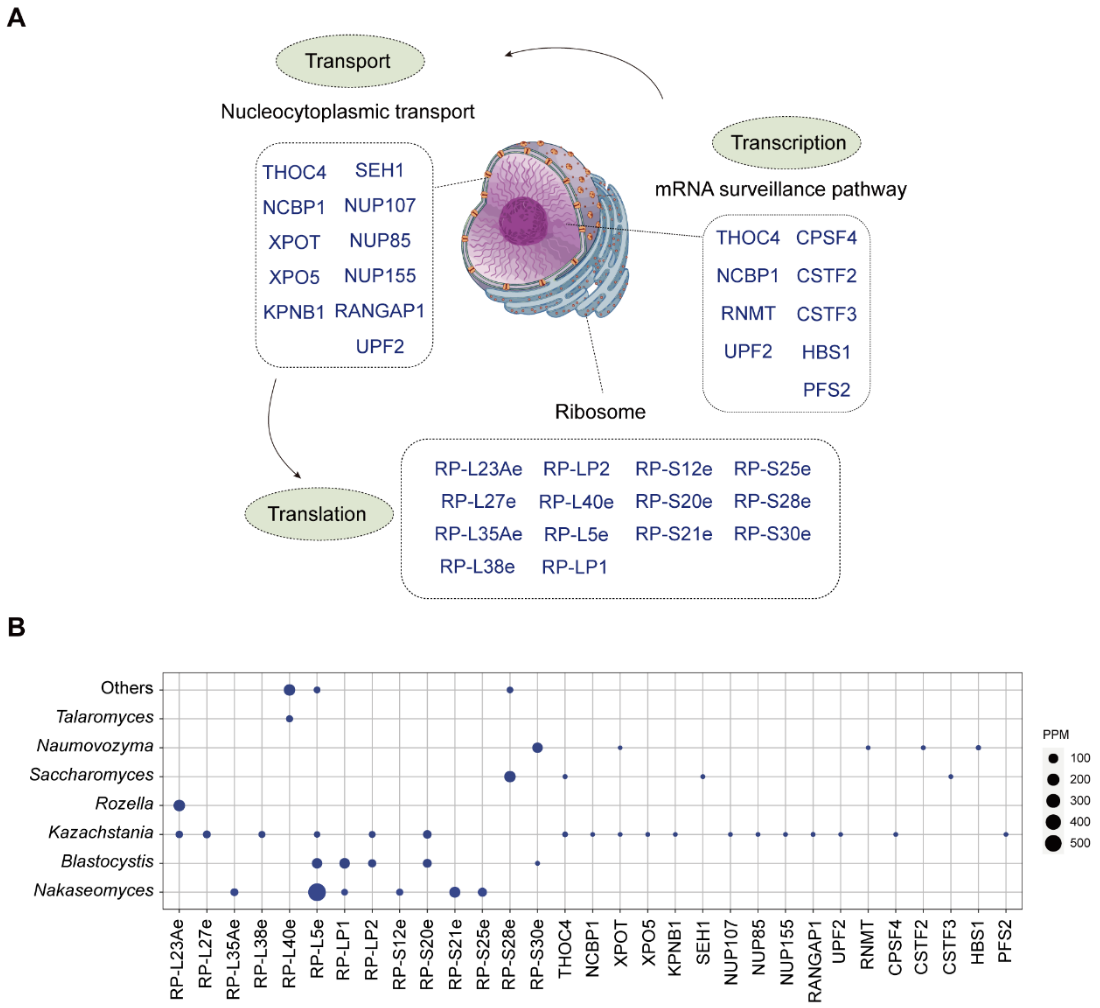

FIG S2 (**A**) PEC weakened expressions of fungal genes involved in mRNA surveillance pathway, nucleocytoplasmic transport and ribosome in pig colon according to metatranscriptomic information. Blue font indicated downregulated KO genes. (**B**) Phylogenetic distribution of sequences in KO genes related to mRNA surveillance pathway, nucleocytoplasmic transport and ribosome assigned to the identified genera. The circle size means the relative abundance (parts per million, PPM) of genes.

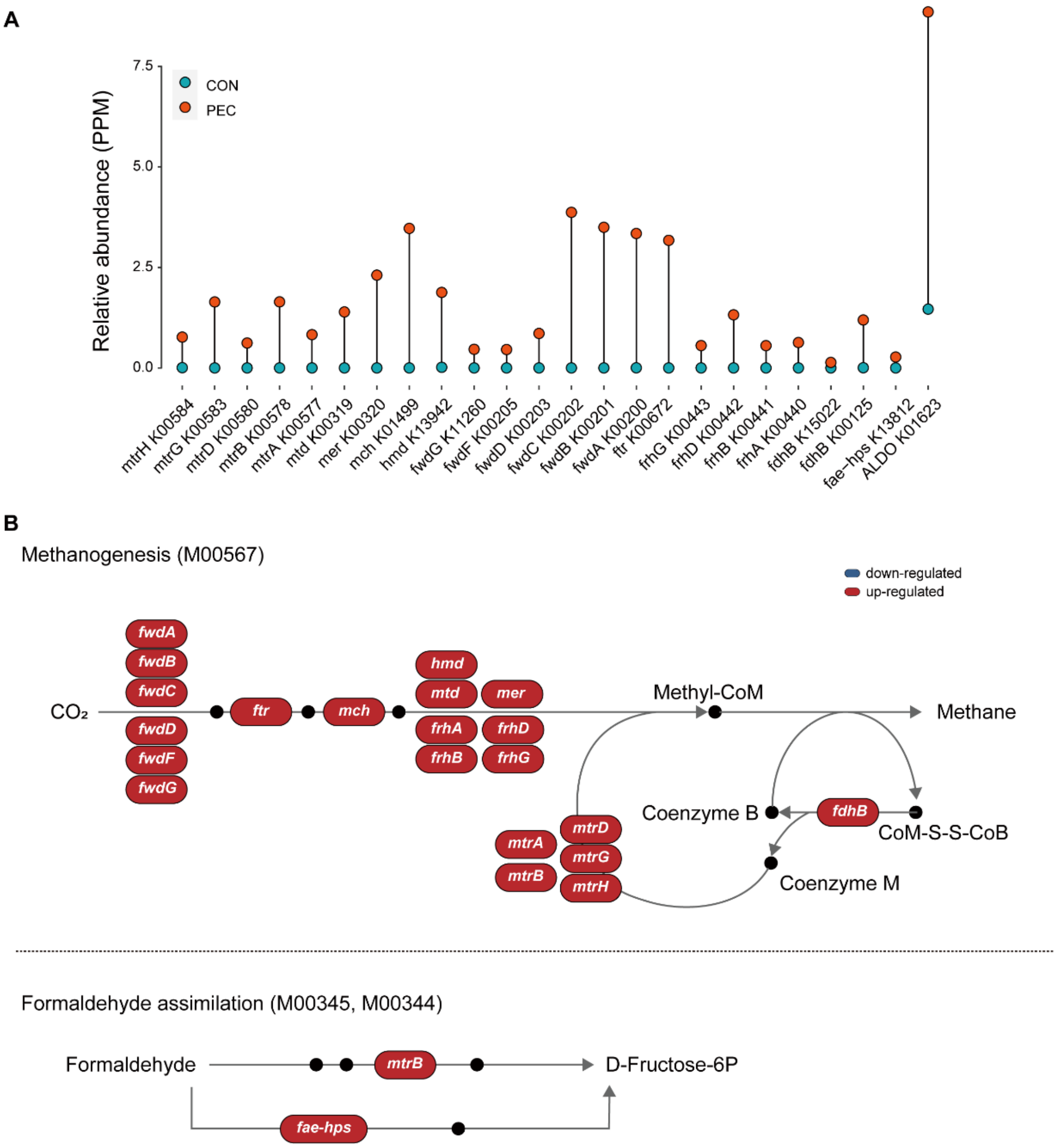

FIG S3 Microbial functions and genera involved in methane metabolism in the CON and PEC groups. (**A**) The dumbbell pot shows the relative abundance of KO genes related to the methane metabolism pathway of pigs in two groups (*n* = 4 per group). (**B**) Metabolic routes for methanogenesis (M00567) and formaldehyde assimilation (M00345 and M00344). The ellipses' color represents two kinds of regulation by pectin-enriched diet. Blue, down-regulation and red, up-regulation.

Supplementary Information

1. **Physico-chemical characteristics analysis according to the methods of GB25533-2010 (1)**

1.1 Loss on drying: Take a flat weighing bottle made of glass into a 105 °C drying oven with the cap on the side of the bottle, heat it for 1 h, then take out and cover it. It was put into a dryer and cooled for 0.5 h, and weighed, and repeat drying until the mass difference between the two times was less than 2 mg. The constant weight was recorded as m_0. 5 g samples were put into the weighing flask. After adding the cover and weighing as m_1, place it in a 105 °C drying oven with the bottle cap on the side of the bottle. After drying for 2 h ~ 4 h, take it out and put it in a dryer for cooling for 0.5 h. Then weigh it and then put it in a drying oven for drying for 1h. And repeat the above operation until the mass difference between the two times was not more than 2 mg, which was the constant weight m_2. The loss on drying (X_1) of pectin was calculated according to Eq. 1.1:

$$X_1 = \frac{m_1 - m_2}{m_1 - m_0} \times 100\%$$

1.2 Acid infusible ash: 2 g pectin (m_1) was slowly heated to complete carbonization with a low fire, then placed in a high temperature furnace and burned to a constant weight at 550 °C. Transfer the burning residue to a 50 mL beaker, slowly add 20 mL of hydrochloric acid solution, boil for 5min, and drain with a sand core filter (m_2), wash with hot water until the filter did not contain chloride ions, and then dry in a drying oven at 105 ± 2 °C to a constant weight (m_3). The acid infusible ash (X_2) of pectin was calculated according to Eq. 1.2:

$$X_2 = \frac{m_3 - m_2}{m_1} \times 100\%$$

1.3 GalA content and DM: **1)** 5g pectin was weighed and placed in a beaker. 100 mL

hydrochloric-ethanol solution was added and stirred for 10 min, and then filtered by a G3 core funnel which was dried to constant weight (m_0). After vacuum suction and filtration, the filter was washed with hydrochloric-ethanol solution for 6 times (15 mL/time), and then washed with ethanol solution for several times until the filter did not contain chloride ion. Finally, the filter was washed with 20 mL absolute ethanol and dried at 105°C for 2h. After cooling, the filter was weighed and recorded as m_1. **2)** 10% of the dried sample was accurately weighed and transferred into a 250 mL corked conical flask and moistened with 2 mL absolute ethanol. Add 100 mL of newly boiled and cooled water, cover the bottle stopper, rotate until the sample was completely dissolved, add 5 drops of phenolphthalein indicator, titrate with 0.1mol/L sodium hydroxide standard titration solution, titrate to pink 30s without fade as the end point, and record the volume V_1 (initial titration degree) of the consumed 0.1 mol/L sodium hydroxide standard titration solution. Add 20.0 ml of 0.5 mol/L sodium hydroxide solution, cover the bottle stopper, shake vigorously and stand for 15 min, add 20.0 ml of 0.5 mol/L hydrochloric acid standard titration solution, shake until the pink disappears, then titrate with 0.1 mol/L sodium hydroxide standard titration solution, shake vigorously until the end point that the weak pink for 30s without fading. The volume V_2 (saponification titration) of 0.1 mol/L sodium hydroxide standard titration solution consumed was recorded. The GalA content (X_3) and DE (X_4) of pectin was calculated according to Eq. 1.3.1 and Eq. 1.3.2, respectively:

Eq. 1.3.1

$$X_3 = \frac{19.41 \times (V_1 + V_2)}{\frac{1}{10}(m_1 - m_0)} \times 100\%$$

Eq. 1.3.2

$$X_4 = \frac{V_2}{V_1 + V_2} \times 100\%$$

1.4 SO_2 and Pb: The determinations of SO_2 and Pb referred to GB 5009.34-2016 (2) and GB 5009.12-2017 (3), respectively.

2. Metabolome analysis

2.1 Sample treatment**:** Ileal contents and feces samples were slowly thawed at 4°C, and a 50-mg aliquot was taken to mix with 800 μL of methanol. The mixture was vortexed for 30 s and maintained at -40°C for 60 min, followed by centrifugation at 12,000 ×rpm for 15 min at 4°C.

2.2 Conditions of chromatographic separation: The column temperature was maintained at 40°C. The mobile phase consisted of mobile phase A (water plus 5% [vol/vol] acetonitrile and 0.1% [vol/vol] formic acid) and mobile phase B (acetonitrile plus 0.1% [vol/vol] formic acid) at a flow rate of 0.3 mL/min. The elution procedure was as follows: 5% mobile phase B from 0 to 1 min, 5% to 95% mobile phase B from 1 to 11 min, and 95% to 5% mobile phase B from 11 to 19.5 min. The injection volume was 3 μL, and the autosampler was maintained at 4°C.

Reference:

(1) GB 25533-2010 Food Additives Pectin[S]. 2010(in Chinese).

(2) GB 5009.34-2016 Determination of Sulfur Dioxide in Food Stuffs[S]. 2016 (in Chinese).

(3) GB 5009.12-2017 Determination of Lead in Food [S]. 2017(in Chinese).
